# Plasma Vitamin C Concentrations and Cognitive Function: A Cross-Sectional Study

**DOI:** 10.3389/fnagi.2019.00072

**Published:** 2019-04-02

**Authors:** Nikolaj Travica, Karin Ried, Avni Sali, Irene Hudson, Andrew Scholey, Andrew Pipingas

**Affiliations:** ^1^Centre for Human Psychopharmacology, Swinburne University of Technology, Melbourne, VIC, Australia; ^2^National Institute of Integrative Medicine, Hawthorn, VIC, Australia; ^3^Discipline of General Practice, The University of Adelaide, Adelaide, SA, Australia; ^4^Health and Sports Institute, Bond University, Gold Coast, QLD, Australia; ^5^School of Science, College of Science, Engineering and Health, Department of Mathematical Sciences, Royal Melbourne Institute of Technology (RMIT), Melbourne, VIC, Australia; ^6^School of Mathematical and Physical Sciences, The University of Newcastle, Callaghan, NSW, Australia

**Keywords:** vitamin C, ascorbic acid, cognition, total recall, attention, central nervous system

## Abstract

Vitamin-C is a water soluble molecule that humans have lost the ability to produce. Vitamin-C plays a role in CNS functions such as neuronal differentiation, maturation, myelin formation and modulation of the catecholaminergic systems. A recent systematic review by our team indicated the need for further research into the relationship between plasma vitamin C and cognition in cognitively intact participants using plasma vitamin C concentrations instead of estimates derived from food-frequency-questionnaires (FFQ), and more sensitive cognitive assessments suitable for cognitive abilities vulnerable to aging. It was hypothesized that higher plasma vitamin C concentrations would be linked with higher cognitive performance. This cross-sectional trial was conducted on healthy adults (*n* = 80, Female = 52, Male = 28, 24–96 years) with a range of plasma Vitamin C concentrations. Cognitive assessments included The Swinburne-University-Computerized-Cognitive-Assessment-Battery (SUCCAB) and two pen and paper tests, the Symbol-Digits-Modalities-Test (SDMT) and Hopkins-Verbal-Learning-Test-Revised (HVLT-R). The pen and paper assessments were conducted to establish whether their scores would correlate with the computerized tasks. Plasma-Vitamin C concentrations were measured using two biochemical analyses. Participants were grouped into those with plasma vitamin-C concentrations of adequate level (≥28 μmol/L) and deficient level (<28 μmol/L). The SUCCAB identified a significantly higher performance ratio (accuracy/reaction-time) in the group with adequate vitamin-C levels vs. deficient vitamin-C on the choice reaction time (*M* = 188 ± 4 vs. 167 ± 9, *p* = 0.039), immediate recognition memory (*M* = 81 ± 3 vs. 68 ± 6, *p* = 0.03), congruent Stroop (*M* = 134 ± 3 vs. 116 ± 7, *p* = 0.024), and delayed recognition tasks (*M* = 72 ± 2 vs. 62 ± 4, *p* = 0.049), after adjusting for age (*p* < 0.05). Significantly higher scores in immediate recall on the HVLT-R (*M* = 10.64 ± 0.16 vs. 9.17 ± 0.37, *p* = 0.001), delayed recall (*M* = 9.74 ± 0.22 vs. 7.64 ± 0.51, *p* < 0.001), total recall (*M* = 27.93 ± 0.48 vs. 24.19 ± 1.11, *p* = 0.003) were shown in participants with adequate plasma Vitamin-C concentrations, after adjusting for vitamin-C supplementation dose (*p* < 0.05). Similarly, higher SDMT scores were observed in participants with adequate plasma Vitamin-C concentrations (*M* = 49.73 ± 10.34 vs. 41.38 ± 5.06, *p* = 0.039), after adjusting for age (*p* < 0.05). In conclusion there was a significant association between vitamin-C plasma concentrations and performance on tasks involving attention, focus, working memory, decision speed, delayed and total recall, and recognition. Plasma vitamin C concentrations obtained through vitamin C supplementation did not affect cognitive performance differently to adequate concentrations obtained through dietary intake.

**Clinicaltrials.gov Unique Identifier:** ACTRN 12615001140549, URL: https://www.anzctr.org.au/Trial/Registration/TrialReview.aspx?id=369440.

## Introduction

Because humans are unable to synthesize their own vitamin C they are particularly vulnerable to vitamin C deficiency (Hansen et al., 2014). Based on population averages, plasma concentrations <28 μmol/L are deemed to be deficient or marginally deficient, and ≥28 μmol/L is considered adequate (Hampl et al., 2004).

Although scurvy is considered to be relatively rare in Western populations, vitamin C deficiency is the fourth most prevalent nutrient deficiency reported in the United States (Schleicher et al., 2009). Reports from cross-sectional population surveys (Hampl et al., 2004; Schleicher et al., 2009) have consistently estimated that at least 10–15% of the adult population in the western world suffer from deficient vitamin C levels, with highest rates (30%) arising in the elderly (Hsiao et al., 2013), with some requiring hospitalization (Zhang et al., 1999; Harrison, 2012). The recent New Zealand CHALICE study (Pearson et al., 2017) revealed suboptimal levels in 62% of 400 healthy 50 year olds, and either a marginal or severe deficiency in 15.4% of the cohort.

Vitamin C blood concentrations are transient, with a number of factors affecting circulating concentrations (Lecomte et al., 1994; Brubacher et al., 2000; Padayatty et al., 2007; Fukushima and Yamazaki, 2010). Factors relating to plasma vitamin C depletion include malabsorption and certain acute and chronic diseases (Hoffman, 1985; Deicher and Hörl, 2003). Some medical conditions can reduce the absorption of vitamin C and/or increase the amount needed by the body. Low vitamin C concentrations have been associated with gastrointestinal disease, liver disease, cancer, asthma, and diabetes. Pregnancy also affects ascorbate levels, with lactation potentially leading to significant losses of maternal vitamin C.

One group particularly vulnerable to vitamin C deficiency are the elderly (>60 years) (Iqbal et al., 2004). Vitamin C levels in tissues, such as the brain and muscle are reduced with age to as little as 25% of those found in children (Lewin, 1976). This may be explained by increased reactive oxygen species [ROS and/or reduced ascorbate recycling in cells, as a result of reductions in the production of glutathione- a molecule required for recycling vitamin C (Rizvi et al., 2009)]. Lowered tissue levels with age have also been attributed to reduced fruit and vegetable consumption in the elderly (Newton et al., 1985; Iqbal et al., 2004).

A basic principle of nutrition is that most nutrients have a non-linear, inverted U-shaped association with optimum physiological function. In order to maintain optimal health, it is becoming increasingly acknowledged that vitamin C is required at concentrations (adequate/optimal) above those needed for the prevention of scurvy (severe depletion) (Carr and Frei, 1999). The category of marginal vitamin C deficiency can rarely be singled out from severe and marginal vitamin C deficiency. Some of the earliest signs of deficiency include fatigue, as deficiency progresses, individuals are known to present with severe fatigue, confusion, and depression (Basu and Donaldson, 2003).

Symptoms of vitamin C deficiency develop after weeks to months of vitamin C depletion (Basu and Donaldson, 2003). Depletion of vitamin C may be linked to a low average intake of <10 mg/d. Usually, ascorbate (reduced form of vitamin) has a half-life between 7 and 14 days in plasma, however, if ingestion stops completely, and levels drop below the sub-baseline, the half-life is significantly longer, approximately 35–40 days (Kallner et al., 1979; Noble et al., 2013). Organs such as the brain are particularly resilient to vitamin C depletion due to ascorbate being recycled by glutathione in astrocytes through the pentose phosphate pathway (Harrison and May, 2009). As plasma concentrations decline, more ascorbate is pumped into the cerebral spinal fluid in order to maintain homeostasis (Bowman et al., 2009). Duration of deficiency has been shown to influence brain ascorbate concentrations to a higher degree than the amount of depletion (Hornig, 1975). This is exemplified by observations in acute scurvy where brain concentrations of ascorbate are maintained through depletion of peripheral tissues, whereas marginal deficiency for longer periods of time has shown greater brain ascorbate depletions (27% depletion in 14 days in guinea pig brain tissue) (Spector, 1977).

The brain’s ability to recycle vitamin C and neurological symptoms that present from vitamin C deficiencies are reflections of the importance that the vitamin plays in central nervous functioning. The biological mechanisms/processes of vitamin C on brain and neuronal functioning have been well established (Travica et al., 2017). It is a vital co-factor in numerous processes such as the biosynthesis of collagen, carnitine, tyrosine, peptide hormones as well as myelin (Kocot et al., 2017). Vitamin C plays a crucial role in neurotransmission and neuronal maturation and functions (Eldridge et al., 1987). Ascorbic acid acts as a co-factor in the synthesis of neurotransmitters, particularly of catecholamines-dopamine and norepinephrine (Figueroa-Méndez and Rivas-Arancibia, 2015). Vitamin C is suggested to influence this process via modulating the binding of neurotransmitters to receptors and regulating their release (Rebec and Pierce, 1994). Vitamin C is also a cofactor for tryptophan-5-hydroxylase required for the conversion of tryptophan to 5-hydroxytryptophan in serotonin production (Gupta et al., 2014). Vitamin C deficiency has shown to decrease serotonin metabolites in both the cortex and striatum (Ward et al., 2013).

Moreover, ascorbic acid modulates the activity of excitatory receptors such as *N*-methyl-D-aspartate as well as inhibitory receptors such as aminobutyric acid (GABA) (Covarrubias-Pinto et al., 2015) and inhibits the binding of glutamate to the NMDA receptor, thus demonstrating a direct effect in preventing glutamate excitotoxicity which causes neuronal dysfunction and degeneration (Harrison and May, 2009).

Additionally, vitamin C has been shown to be involved in the biosynthesis of L-carnitine (Rebouche, 1991) playing a role in the transport of fatty acids into mitochondria for energy production. Enzymes can readily convert carnitine to acetyl-carnitine and vice versa, according to the metabolic needs of the cell (Mendelson, 2008). Acetyl-carnitine can cross the blood brain barrier and act as a precursor in the production of acetylcholine and supports healthy cerebral blood flow (White and Scates, 1990).

Vitamin C also plays a role in the modulation of neuronal metabolism by changing the preference for lactate over glucose as the primary energy source in neurons sustaining synaptic activity (Castro et al., 2008). Vitamin C is also involved in collagen synthesis in the brain (Harrison et al., 2009). It hydroxylases the amino acids proline and lysine and creates hydroxyproline which is found abundantly in collagen. Collagen is needed for blood vessel formation and integrity (Hansen et al., 2014). Ascorbate-dependent collagen synthesis has also been linked to the formation of myelin sheaths and regeneration of damaged sheaths that surround many nerve fibers (Guo et al., 2018). According to a study using mice deficient in an ascorbate transporter, the concentration of ascorbate in the brain was shown to be below a detectable level. The animals died due to capillary hemorrhage in the penetrating vessels of the brain (Sotiriou et al., 2002).

Due to a high level of poly-unsaturated fatty acids, combined with high rates of cellular metabolism of oxygen, glucose etc., the brain is particularly vulnerable to creating reactive oxygen species (Uttara et al., 2009). Ascorbic acid acts directly by scavenging reactive oxygen and nitrogen species produced during cell metabolism, and further recycles vitamin E involved in cell membrane integrity (Harrison and May, 2009). Additionally, vitamin C has been found to induce the expression of brain derived neurotrophic factor (BDNF) – a component of several survival pathways (Grant et al., 2005).

Alongside vitamin C, a number of nutrients have been shown to affect cognitive function (Gómez-Pinilla, 2008). Examples of these include magnesium (Slutsky et al., 2010), vitamin E (Kang et al., 2006), vitamin D (Garcion et al., 2002), selenium (Chen and Berry, 2003), and Vitamin B12. Amongst these nutrients, Vitamin B12 has been shown to be critical for cognitive function in several research trials, and will therefore be considered a covariate in the analysis of cognitive assessments (Kennedy, 2016). The effects of vitamin B12 (cobalamin), in particular on the central nervous system have been well documented. Vitamin B12 is a water-soluble vitamin required for red blood cell formation, neurological function, and DNA synthesis. More recent research has demonstrated a link between plasma vitamin B12 concentrations and cognitive function, and the potential to reduce the rate of brain atrophy with high dose B-vitamin supplementation that includes B12 (Riggs et al., 1996; Reay et al., 2013). Vitamin B12 deficiency has been linked to psychiatric disorders, including impaired memory, irritability, depression, and dementia (Lindenbaum et al., 1988). Neurological disorders due to vitamin B12 deficiency typically occur in both genders between the ages of 40 and 90 years, with a peak between 60 and 70 years (Healton et al., 1991; Savage and Lindenbaum, 1995; Kennedy, 2016).

A recent review demonstrated higher mean vitamin C concentrations in the cognitively intact groups of participants compared to cognitively impaired groups (Travica et al., 2017). One major limitation conducted in studies with healthy samples was the lack of sensitive cognitive assessments suitable for cognitively intact adults. Qualitative analysis of these studies that used a variety of cognitive assessments in the healthy population revealed a potential association between plasma vitamin C concentrations and cognition.

Additionally, a number of limitations have arisen in the measurement of vitamin C levels in a number of previous studies (Jama et al., 1996; Beydoun et al., 2015). The dietary assessments used have reliability and validity issues as a result of potential recall errors with self-report (Weinstein et al., 2001). Even when food consumption is recalled correctly, differences in storage and cooking can decrease the vitamin C level in the food and absorption can vary amongst participants (Weinstein et al., 2001). Given these issues, dietary self-reports do not necessarily reflect the vitamin C potential for biological action.

While blood samples are a more reliable measure of vitamin C status than self-reported vitamin C intakes, many factors can contribute to the instability of ascorbic acid in biological samples such as heat, light, and elevated pH (acidity). A number of previous studies failed to thoroughly explain blood sample handling and biochemical analysis, underestimation of vitamin C concentrations could occur if samples were not handled properly (Harrison, 2012).

Discriminating between the concentrations of those supplementing on vitamin C from those not supplementing has been an issue in previous studies. The cognitive significance of marginal and severe plasma vitamin C deficiency in comparison to adequate levels has not been thoroughly investigated. In most studies, upper and lower tertiles, quartiles, or quintiles are compared, making it difficult to compare groups between studies. Additionally, the use of dietary vitamin C supplementation has either been ignored in previous research or plasma vitamin C concentrations were not measured in those supplementing with vitamin C in randomized controlled and prospective trials. This may have compromised previous outcomes, as vitamin C supplementation may influence variances in plasma vitamin C concentration and may be complemented with the supplementation of other nutrients which are involved in brain function.

The present study was devised with the aim of exploring whether there is an association between plasma vitamin C concentrations and cognitive function in cognitively intact adults, using paper and pen and computerized cognitive assessments. We hypothesized that there would be a positive correlation between plasma vitamin C concentrations and cognitive performance and that those presenting with adequate plasma vitamin C concentrations would demonstrate higher cognitive performance than those displaying deficient (marginal/severe) plasma vitamin C concentrations. Furthermore, the relationship between vit C and cognition was investigated in participants who did not report vitamin C supplementation and in a larger group that included vitamin C supplementers.

## Methods

### Recruitment

This study was approved by the Human Research Ethics Committees at National Institute of Integrative Medicine and Swinburne University of Technology, and the trial was registered with the Australian New Zealand Clinical Trial Registry (ACTR12615001140549). Participants provided informed written consent.

Cognitively intact adults (>18 years) were sought to participate in this cross-sectional study investigating the association between plasma vitamin C concentrations and cognition. Asymptomatic participants were primarily recruited from The National Institute of Integrative Medicine (NIIM). We included participants displaying no major neurodegenerative condition, i.e., dementia (3MS score >79), and included those likely to be displaying a range of plasma vitamin C concentrations, i.e., varying diets, supplementation, age groups. Participants were excluded if they were pregnant or lactating, color blind, or taking antidepressants, antipsychotics, anxiolytics, illicit drugs or any cognitive enhancing drugs. Participants were also excluded if they were not able to give informed consent.

### Cognitive Assessments

A number of cognitive assessments were performed. These included the Modified Mini Mental State Examination (3MS) (Tombaugh and McIntyre, 1992), Revised Hopkins Verbal Learning Test (HVLT-R) (Brandt, 1991), Symbol Digits Modalities Test (SDMT) (Sheridan et al., 2006) and the Swinburne University Computerized Cognitive Assessment Battery (SUCCAB) (Pipingas et al., 2010).

#### Modified Mini Mental State Examination (3MS)

Participants were initially screened for cognitive impairment using the 3MS. This was implemented as a valid and reliable screening test for the purpose of evaluating major cognitive impairment (scale 0–100).

#### HVLT-R/SDMT (Paper and Pen Tests)

The HVLT-R is a validated paper and pen test designed to examine verbal learning, immediate and delayed recall, and delayed recognition. Participants were required to recognize and recall a list of 12 words immediately (across 3 trials) and after 40 min (4th trial). Delayed recognition was assessed by reading a longer list of words to participants (24 words) and having them respond with a ‘yes’ or ‘no’ if they recognized the word from the original list of 12 words. The 4th HVLT-R word recall trial and delayed recognition were completed after the SUCCAB. Trials 1–3 were scored out of 12 points, delayed recall (trial 4) was also scored out of 12 points and total recall was calculated from the sum of total correct responses for trials 1, 2, and 3 (out of 36 points). Recognition index was scored out of 12 for every correct word recognized.

The symbol digits modalities test (SDMT) (Sheridan et al., 2006) required participants to pair numbers with geometric figures, primarily assessing divided attention, tracking and visual screening. Participants were presented with a key consisting of numbers between 1 and 9 with a corresponding symbol under each number. The test consisted of rows of random symbols with blank squares below each symbol, and participants were given 90 s to fill in the blank squares with the corresponding number. Test scores ranged between 0 and 110.

#### Swinburne University Computerized Cognitive Assessment Battery (SUCCAB)

The SUCCAB is a validated cognitive test battery consisting of eight computer-based cognitive tasks assessing various aspects of cognitive performance (Pipingas et al., 2010). Participants were asked to respond as quickly and accurately as possible in each task. A 4-button response box was used to complete the tasks, with buttons corresponding to task specific response dimensions: color (red, blue, green, or yellow), ‘yes’ or ‘no,’ or the spatial location of objects on the screen (top, bottom, left, or right). The eight tasks that comprised the SUCCAB battery are described in Table 1 below.

**Table 1:** SUCCAB tasks and assessments.

Task name	Task	Cognitive component assessed
Simple reaction time	Pressing the yes button upon seeing a white square.	General alertness/motor speed
Choice reaction time	Pressing of either the blue or red buttons corresponding with blue triangle or red square.	Visual perception decision time
Immediate recognition memory	‘Yes’ or ‘no’ buttons, signaling whether or not they had just seen an abstract image during the earlier viewing period.	Non-verbal recognition memory
Congruent Stroop	Responding by pressing the color button (red, blue, green, or yellow) that corresponded to the print color of the presented word.	Executive functioning and inhibition
Incongruent Stroop	Conducted in the same way as congruent Stroop color word, except that the stimulus words (red, yellow, blue, or green) appeared in print color incongruent with the written word.	Executive functioning and inhibition
Spatial working memory	Multiple trials consisting of a 4 × 4 white grid displayed against a black background, with initially six of the grid positions filled with solid white squares for a brief moment. All filled squares were then removed and only one square was filled. Participants responded with either the ‘yes’ or ‘no’ buttons indicating if the new squares were presented in the same locations as the original set.	Spatial information in working memory
Contextual recognition memory	Objects were presented momentarily one after the other at various locations (top, bottom, left, or right) on the screen. Each of the objects was then presented in the center of the screen. Participants responded using either the top, bottom, left, or right buttons to indicate the image’s original location on the screen.	Episodic memory
Delayed recognition memory	A follow-up task to the immediate recognition memory task tested longer term memory retention of abstract images. Participants were again required to respond ‘yes’ or ‘no’ as to whether or not they had viewed the abstract images earlier.	Recognition memory

Participants initially completed a 10-min practice of the SUCCAB with the experimenter present, explaining and observing the participant during practice tasks. Following the practice run, and once participants were familiar and comfortable with the tasks, they completed the 30 min SUCCAB.

### Plasma Vitamin C Assessment

Fasting (8–12 h) blood tests were performed by the researcher/phlebotomist immediately after the completion of the SDMT. Blood was collected in 2, 6 ml lithium heparin vacutainer blood tubes. The blood tubes were immediately wrapped in foil and kept away from light. Each blood tube was spun in a centrifuge at 3,600 rpm. Plasma from the heparin tubes was aliquoted into separate 3 ml tubes. These were also wrapped in foil and kept away from light. Blood vitamin C concentrations were analyzed using two different biochemical analyses. An additional aim of the study was to compare and verify the effectiveness of an analysis conducted immediately at The National Institute of Integrative Medicine with one conducted by an external laboratory. This was done to ensure that the most reliable blood plasma vitamin C concentrations were used in analyses.

(a) Colorimetric analysis (Chung et al., 2001) is a method of determining the concentration of a chemical element or chemical compound in a solution with the aid of a color reagent. Ascorbic testing was conducted using the Ascorbic acid Assay Kit II (Sigma-Aldrich). In the assay, ascorbic acid concentration was determined using Ferric reducing/antioxidant and ascorbic acid assay. In the assay, Fe^3+^ is reduced to Fe^2+^ by antioxidants present in the sample which results in a colorimetric (593 nm) product. The color reaction is preceded by a reaction catalyzed by an enzyme. The addition of ascorbate oxidase to parallel samples oxidizes any ascorbic acid present allowing for the measurement of the ascorbic acid concentration. This analysis was performed in the NIIM lab using a Sigma-Aldrich kit and SoftMax^^®^^ Pro Software on the EMax^^®^^ Plus Microplate Reader. This analysis was run within 30 min of the blood being taken.
(b) A high performance liquid chromatographic (HPLC) (Chung et al., 2001) method was also used for the quantitation of vitamin C in plasma samples. The analysis was conducted 2–3 days following the blood test. The blood was spun in a centrifuge at 1,500 rpm, plasma separated and transported on dry ice (-80°C) and kept away from light. The HPLC testing was done by Sullivan Nicolaides Pathology (Queensland, Australia) using the Chromsystems^^®^^ (Gräfelfing, Germany) Vitamin C Plasma/Serum HPLC kit. Level I and II Lyophilized quality vitamin C plasma controls were used. 100 μl of sample/calibrator/control was pipetted into a labeled light protected reaction vial. 100 μl of the internal Standard was added and mixed for 30 s (vortex). This was then centrifuged for 5 min at 15,000 × *g* and 20 μl of the supernatant was injected into the HPLC system. The HPLC analysis was carried out using a Waters 2695 Separations Module equipped with a dual wavelength UV detector set to 245 nm. The column temperature was maintained at 25°C. The generated chromatogram was used to determine vitamin C concentrations. Blood was also taken in a sodium citrate vacutainer tube for analysis of serum Vitamin B12 through Melbourne Pathology. A vitamin B12 immuno assay (Roche E602) was used to measure total vitamin B12 concentrations. This assay employs the competitive inhibition enzyme immunoassay technique (Lee and Griffiths, 1985). This was analyzed within 3 days of blood being taken.

### Questionnaires

Alongside assessing basic demographic information, a questionnaire assessed intake of prescribed medications, dietary supplementation (dose/frequency), smoking status, highest level of education, exercise (duration/type), family history of neurodegenerative disease, and any history of an incident possibly contributing to cognitive dysfunction. Exercise was defined as Moderate (50–65% of maximum heart rate) or vigorous (70–85% of maximum heart rate) (Swain and Franklin, 2006).

A long term (1 year) dietary intake of a number of additional nutrients was assessed using the computerized version of the dietary questionnaire for Epidemiological Studies, Version 2 (DQES v2) (Ireland et al., 1994). The Cancer Council DQES v2 covered five types of dietary intake based on the previous 12 months, incorporating 80 items: cereal foods, sweets and snacks, dairy products, meats and fish, fruit and vegetables and alcoholic beverages. It summarizes the intake of key nutrients all vital for CNS function such as magnesium, Vitamin E, selenium, folate, etc.

Secondly, an in-house developed short-term food frequency questionnaire (FFQ) was administered. This FFQ (based on the Cancer Council FFQ) assessed specifically what the consumption of vitamin C and vitamin B12 rich foods was within 2 weeks prior to the testing session.

### Mood

Mood was assessed as a potential confounder to cognitive performance. The Bond Lader Mood questionnaire (Bond and Lader, 1974) is designed to measure four different concepts of mood: Mental Sedation or intellectual impairment, physical sedation or bodily impairment, tranquillization or calming effects, other types of feelings or attitudes. The scales comprise of 16, 10 cm lines anchored at each end by words descriptive of opposing statements (bipolar). On this linear scale, the participant indicated their mood by placing a mark between the 2 opposite words on either side. High reliability and validity have been demonstrated with this assessment. From the resultant scores, three derived measures can be isolated. These have been described as representing the following: alertness, calmness, and contentedness. Scores for each measure represent the unweighted average number of millimeters (maximum 100 mm) from the negative antonym for the individual scales contributing to the measure. We also added 2 scales from the cognitive demand battery that we implemented in our previous research (Massee et al., 2015). These scales assessed exhaustion/energy and stress/calmness and were measured the same way as the Bond Ladder scales.

### Procedure

Participants were asked to fast between 8–12 h prior to attending the testing session. They were also asked to refrain from any exercise on the morning of the testing session. Firstly, the demographic/history questionnaire and mood assessment was administered and followed by the 3MS assessment. The 3MS was followed by the FFQ, which assessed vitamin C and Vitamin B12 intake specifically. Following the FFQ, the HVLT-R cognitive assessment was administered. Participants then undertook the 10-min practice run of the SUCCAB before the commencement of the 30 min SUCCAB test battery. Following the SUCCAB, the SDMT and the delayed recognition and recall of the HVLT-R were then administered. The second FFQ (DQES v2) proceeded these cognitive tests and was followed by the blood testing and colorimetric analysis. Plasma was aliquoted and foiled for the biochemical analyses and specifically delivered on dry ice to the pathology company for HPLC analysis.

### Sample Size Calculation

There had been no previous study exploring the effects of plasma vitamin C concentrations specifically using the SUCCAB and therefore no available data that could be used in a power analysis calculation. A recent study established aged-related mean SUCCAB values for each task in a sample of cognitively intact adults (*n* = 120, mean age = 53, *SD* = 16 years) (Pipingas et al., 2010). Results indicated significant differences for every 10 years of age of up to 100 ms (1 SD) on the spatial working memory task. Given the sensitivity of this task to cognitive aging, this is a useful tool in aging research to measure the degree of cognitive decline and to examine the efficacy of interventions. A sample size calculation indicated that 80 participants was sufficient to detect a significant mean difference of 100 ms (*SD* = 211) on the spatial working memory reaction time task in the SUCCAB, with 80% power and 95% confidence. This difference relates to 10 years of cognitive aging.

### Statistical Analyses

Analyses were performed using SPSS IBM statistics version 23 package. Statistical significance was set at *p* < 0.05. Values that were 2 standard deviations away from their means for the cognitive assessments were excluded from analysis. A Bonferroni correction adjustment was used in the ANOVA and ANCOVA analyses to avoid committing a type 1 error in the analyses. Descriptive analyses were conducted on all variables including mean dietary intakes, supplement doses, exercise etc.

A Bland–Altman analysis (Giavarina, 2015) was used to determine whether the two plasma vitamin C biochemical methods provided consistent results. This analysis involved correlating the difference (T-S) between the test (T = Colorimetric analysis) and comparative method (S = HPLC) against the average (T+S)/2 of the results obtained from the two methods. A Bland–Altman plot was also used to show the difference between the two methods against the average of both methods, the confidence intervals being displayed. Additionally, a Spearman’s correlation was conducted to determine the correlation between the two biochemical methods. As recommended by Bland and Altman, comparability was achieved if 95% of the data points were within ±2 SDs of the mean difference.

If consistency was demonstrated, the concentrations were averaged between the two biochemical analyses. Daily vitamin C consumption (mg/d) based on the food frequency questionnaire was also compared with the plasma vitamin C concentrations using a Spearman correlation.

Vitamin C blood plasma concentrations were correlated with cognitive performance using Spearman rank correlations. Scatterplots and Spearman correlations were used to assess the direction and strength of association between vitamin C concentrations and cognitive performance. This analysis was also used to visualize the impact of vitamin C supplementation on cognition relative to non-supplementers. In order to determine whether those who self-reported vitamin C supplementation would have an effect on cognitive performance, further Spearman correlations were conducted with the exclusion of those supplementing on vitamin C.

Primary analyses were conducted to compare cognitive performance between those in the adequate plasma vitamin C group with those in the deficient group. Average vitamin C concentrations were sub grouped into internationally established reference ranges representing adequate (≥28 μmol/L) or deficient (<28 μmol/L) concentrations. Descriptive statistics were compared between those with adequate versus deficient vitamin C concentrations using an analysis of variance (ANOVA) for continuous variables. A chi-square analysis was performed to assess the association between categorical variables such as gender, smoking status and prescribed medications and the adequate versus deficient status of participants (group effect) on plasma vitamin C concentrations.

For the SUCCAB, mean response time and percentage accuracy were calculated for each task. The accuracy percentage was divided by the mean response time (sec) to give an overall performance ratio score. This ratio also assisted in accounting for the issue of the speed versus accuracy trade-off that exists with increasing age (Starns and Ratcliff, 2010; Forstmann et al., 2011).

Lower reaction times are indicative of faster response times and with higher performance ratios indicating a combination of faster reaction times and greater trial accuracy. Finally, correlational analyses were performed between the scores on both of the paper and pen cognitive tasks and performance on each of the SUCCAB tasks. Cognitive performance was compared between those with adequate versus deficient vitamin C concentrations using an analysis of covariance (ANCOVA) for potential continuous confounder variables. Such potential confounding variables were assessed using the correlation regression analyses generated in the ANCOVA, with covariates included in the ANCOVA only if they had a significant effect (*p* < 0.05) on the cognitive performance variables. Additional ANCOVAs were conducted on cognitive performance between the deficient and adequate groups in which those supplementing on vitamin C were excluded. This was performed to investigate whether there would be a difference in cognitive performance between the groups with the exclusion of vitamin C supplementers.

A number of cognitive variables were derived from the HVLT-R assessment. Trials 1–3, delayed recall (trial 4), total recall, and recognition index were scored based on the number of correct responses. SDMT performance was scored out of 110 points.

## Results

### Participants

The trial was conducted in Melbourne, Australia between November 2016 to January 2018. Participant demographic details are presented in Table 2.

**Table 2:** Participants’ baseline characteristics and mood assessment.

A) Demographics (*n* = 80)	Unit	Mean ±*SD*
Age	Years	60.97 ± 15.76 (range: 24–95)
Gender	m/f	27/53 (33.7%/66.3%)
BMI	kg/m^2^	26.00 ± 3.16
No exercise (*n* = 29)	Minutes/week	0
Moderate exercise (*n* = 37)	Minutes/week	139.46 ± 109.23
Vigorous exercise (*n* = 14)	Minutes/week	202.86 ± 85.79
Education	Years	13.2 ± 1.46
Current smoker	n (Yes/No)	4/76 (5%/95%)
Family history of Neurodegenerative disease	n (Yes/No)	20/60 (25%/75%)

**B) Type of prescription med for condition**		**Sample size *n* (%)**

Prescribed medications		53 (73.75%)
Blood pressure medication		35 (43.75%)
Blood thinners		18 (22.5%)
Statin		10 (12.5%)
Anti-inflammatory meds		5 (6.25%)
Reflux/gut issue meds		5 (6.25%)
Thyroid meds		5 (6.25%)
Other		6 (7.5%)

**C) Mood assessment (Bond–Lader)**	**Range**	**Mean ± *SD***

Alertness	0–100	60.76 ± 14.93
Calmness	0–100	66.04 ± 61.93
Contentedness	0–100	67.26 ± 15.09
Energetic	0–100	57.86 ± 22.69
Stressed	0–100	40.3 ± 23.52

*n, sample size; Med, medication; Moderate exercise, 50–60% of maximum heart rate; Vigorous exercise, 70–85% of maximum heart rate; SD, standard deviation*.

There was a large age range (24–95) amongst the 80 recruited participants, the majority of participants were older (mean: 60.97 years ± 15.76). A majority of the sample was female (66.3%) with an average overweight BMI of 26.00 (*SD* = 3.16). 36.2% of the sample did not undertake any form of exercise 2 weeks prior to testing and a majority of those that did, reported a moderate intensity. Most participants reported no family history of neurodegenerative disease. On average, each of the assessed mood variables displayed mid-range values, indicating no extreme emotional display before the cognitive testing.

The majority (73.75%) of participants were on some form of prescribed medication, specifically anti-hypertensives (43.75%) and blood thinners (22.5%). Daily nutritional supplements are summarized in Table 3.

**Table 3:** Supplements.

Supplement	Number of participants	Mean dose/day ±*SD*
Multi-vitamin	7	Varying doses consisting of multiple vitamins
Vitamin C	20 (including multi vitamin)	860 mg ± 595.06
Vitamin B12	21	210.48 μg ± 240.78
Vitamin D	24	1500 IU ± 456.5
Vitamin K	3	116 mg ± 55.08
Co-enzyme 10	9	127.2 mg ± 32.2
Fish oil/krill oil	15	1,266.7 mg ± 258.2
Magnesium	21	654.5 mg ± 190.33
Zinc	2	12.07 mg ± 2.86
Chromium	2	40 μg ± 10
Flaxseed oil	2	1,000 mg ± 0
Curcumin	5	860 mg ± 219

*IU, international units; mg, milligram; μg, microgram; SD, standard deviation*.

Supplements were only considered if they were taken within 1 week prior to the testing session. For those taking multivitamins, each individual vitamin was considered and contributed to intake. Most supplements included vitamin C, B12, D, magnesium and/or fish/krill oil. For those supplementing with vitamin C, concentrations and frequency of vitamin C supplementation did not vary immensely between participants. Frequent (almost daily) vitamin C supplementation was amongst those demonstrating plasma vitamin C concentrations >100 μmol/l. A majority reported multi-supplementation and not one single mineral/vitamin. This is exemplified by almost all of those supplementing on vitamin C also supplementing on some form of vitamin B12. 48 participants (60%) reported supplementation, with 42% (*n* = 20) of these reporting vitamin C supplementation in particular. Estimated FFQ nutritional intakes are displayed in Table 4.

**Table 4:** Daily nutritional intake.

Nutrient (*n* = 47)	Unit	Mean ±*SD* (*n* = 50)	RDI	% within RDI
Vitamin C (*n* = 80)	mg/d	82.19 ± 23.57	>100 mg/d (Levine et al., 1996)	26.25
Vitamin B12 (*n* = 80)	μg/d	2.88 ± 0.97	2.4 μg/d (Mollin and Ross, 1952)	60
Vitamin A (retinol)	μg/d	268.40 ± 244.50	M: 900 μg/d (Trumbo et al., 2001)	0
			F: 700 μg/d (Trumbo et al., 2001)	
Vitamin E	mg/d	13.89 ± 6.54	15 mg/d (Frei and Traber, 2001)	38.3
Vitamin D	μg/d	3.66 ± 1.73	5–15 μg/d (Holick, 1996)	25.5
Folate	μg/d	433.55 ± 222.62	400 μg/d (O’Keefe et al., 1995)	48.9
Vitamin B1	mg/d	1.56 ± 0.93	M: 1.2 mg/d (Martel and Franklin, 2017)	55.3
			F: 1.1 mg/d (Martel and Franklin, 2017)	
Vitamin B2	mg/d	2.10 ± 0.97	M: 1.3–1.6 mg/d (Iuliano et al., 2013)	80.9
			F: 1.1–1.3 mg/d (Iuliano et al., 2013)	
Vitamin B3	mg/d	23.89 ± 9.44	M: 16 mg/d (Iuliano et al., 2013)	76.6
			F: 14 mg/d (Iuliano et al., 2013)	
Vitamin B5	mg/d	3.38 ± 1.13	M: 6 mg/d (Fox and Linkswiler, 1961)	36.2
			F: 4 mg/d (Fox and Linkswiler, 1961)	
Vitamin B6	mg/d	1.24 ± 0.58	M: 1.3–1.7 mg/d (Madigan et al., 1998)	38.3
			F: 1.3–1.5 mg/d (Madigan et al., 1998)	
Vitamin B7	μg/d	34.36 ± 13.83	30 μg/d (Institute of Medicine (US) Standing Committee on the Scientific Evaluation of Dietary Reference Intakes and Its Panel on Folate, Other B Vitamins, and Choline, 1998)	59.6
Iron	mg/d	12.35 ± 4.51	M: 8 mg/d (Trumbo et al., 2001)	83.0
			F: 8–18 mg/d (Trumbo et al., 2001)	
Mg	mg/d	509.44 ± 209.60	M: 420 mg/d (Greger et al., 1981)	78.7
			F: 320 mg/d (Greger et al., 1981)	
Zinc	mg/d	9.89 ± 3.54	M: 11 mg/d (Trumbo et al., 2001)	59.6
			F: 8 mg/d (Trumbo et al., 2001)	
Omega 3	mg/d	526.39 ± 300.89	M: 160 mg/d (Trumbo et al., 2002)	97.9
			F: 90 mg/d (Trumbo et al., 2002)	
Iodine	μg/d	120.58 ± 46.75	150 μg/d (De Benoist et al., 2008)	23.4
Caffeine	mg/d	532.74 ± 362.11	210 mg/d (Smith et al., 2000)	70.2
Alcohol	g/d	4.93 ± 9.45	10 mg/d (National Health and Medical Research Council, 2009)	85.1

*F, female; M, male; mg/d, milligram per day; μg/d, microgram per day; RDI, recommended daily intake; SD, standard deviation; n, sample size*.

On average, a majority of participants were within the RDI for the various B vitamins. However, close to one quarter met the RDI for vitamins C, A, E, D and iodine. Subgroup analyses (not shown) revealed no significant differences in nutritional intake between genders, age groups, BMI groups and those exercising.

### Plasma Vitamin C Concentrations

The blood plasma vitamin C concentrations for each participant using each of the two biochemical techniques are displayed on Supplementary Figure S1.

Plasma concentrations ranged from 7 to 120 μmol/L. Between the two biochemical analyses, plasma vitamin C concentrations were similar for each participant. The Bland and Altman method (Giavarina, 2015) was used to compare the two methods. Firstly, the difference between the HPLC and colorimetric methods (HPLC - Colorimetric) was correlated with the average (HPLC + Colorimetric)/2 of the two methods. Spearman’s correlation indicated no correlation in this difference and average between the methods [*r*_s_(80) = 0.159, *p* = 0.16]. Furthermore, a Bland–Altman plot (Supplementary Figure S2) graphically demonstrated the difference of the two paired measurements against the mean of the two measurements. As recommended by Bland and Altman, comparability was achieved given 95% of the data points were within ±2 standard deviations of the mean difference (Giavarina, 2015).

Spearman’s correlation revealed a strong significant relationship between the two analyses (*r* = 0.96, *p* < 0.01). Given the high consistencies between the two biochemical analyses using the Bland and Altman method, their means were used for additional cognitive analyses such as correlations.

A majority (*n* = 67, 83.75%) of participants were within the adequate vitamin C reference range (≥28 μmol/L), displaying a mean plasma vitamin C concentration of 53.09 ± 19.77 μmol/L 16.25% (*n* = 13) were within the vitamin C deficiency range (<28 μmol/L), displaying a mean plasma concentration of 16.27 ± 6.45. Those deficient vitamin C were combined into one group and those displaying either adequate or optimal levels were combined into another group. As observed in Supplementary Figure S3, a Spearman correlation revealed a significant relationship between average daily dietary vitamin C intake and the plasma vitamin C concentrations [*r*_s_(80) = 0.438, *p* < 0.001].

Demographic, mood and nutritional differences between the adequate and deficient group using an ANOVA analysis are displayed in Table 5.

**Table 5:** Adequate versus deficient vitamin C group demographics.

	Group 1:		Group 2:
Variable	Adequate vit C levels	*n*	Deficient vit C levels	*n*			χ^2^ test *p*-value
**(A)** Gender *n* % (M/F)	74/89%	20/47	26/11%	7/6			**0.094%**
Prescribed meds %	61.2%	41	92.3%	12			**0.016%**
Smoker %	1.5%	1	23.1%	3			**0.007%**

	**Mean ± *SE***		**Mean ± *SE***		**Mean difference**		***p*-value**
					**Mean**	***SE***	

Age	59.51 ± 1.89	67	68.54 ± 4.25	13	9.03	2.36	0.06
Education (years)	13.23 ± 1.5	67	13.0 ± 1.1	13	0.23	0.4	ns
Exercise – moderate intensity (mins/week)	140.3 ± 20.11	33	132.5 ± 14.36	4	7.8	5.75	ns
Exercise – vigorous intensity (mins/week)	214.17 ± 24.75	12	135 ± 45.0	2	79.17	20.25	ns
**(B) Mood**					
Alertness	60.03 ± 1.91	67	64.62 ± 2.84	13	4.56	0.93	ns
Calmness	58.86 ± 2.07	67	62.15 ± 7.66	13	3.26	5.59	ns
Contentedness	65.87 ± 1.87	67	74.46 ± 3.37	13	8.60	1.5	0.06
Energetic	56.79 ± 2.69	67	63.38 ± 7.27	13	6.59	4.58	ns
Stressed	41.07 ± 2.89	67	36.31 ± 6.46	13	4.77	3.60	ns
**(C) Nutrient intake**							
Dose of Vit C supplementation (mg/d)	860 ± 62.19	20	0	0	860	62.19	***p* < 0.01**
Vit C dietary intake (mg/d)	85.62 ± 2.64	67	74.23 ± 3.25	13	11.39	0.61	ns
Plasma vitamin C (μmol/L) (non-supplementers)	47.32 ± 1.90	47	16.27 ± 1.79	13	28.59	0.11	***p* < 0.01**
Plasma vitamin C (μmol/L) (supplementers)	66.65 ± 5.79	20	Na	0		5.79	Na
Vitamin B12 supplementation (μg/d)	210.4 ± 52.54	21	0	0	210.4	52.54	***p* < 0.01**
Vitamin B12 dietary (μg/d)	2.87 ± 0.19	67	2.96 ± 0.27	13	0.084	0.08	ns
Serum Vitamin B12 (pmol/L)	585.6 ± 42.46	67	342.4 ± 43.81	13	243.2	1.35	**0.016**
Vitamin D supplementation (IU/d)	1520.8 ± 92.66	24	1000	1	520.83	92.66	ns
Vitamin D dietary (ug/d)	3.63 ± 0.29	38	3.32 ± 0.56	9	0.31	0.27	ns
Magnesium supplementation (mg/d)	662.5 ± 42.90	20	500	1	162.5	42.90	ns
Magnesium dietary (mg/d)	508.3 ± 33.78	38	514.5 ± 76.02	9	6.12	42.24	ns
Fish oil/krill oil supplementation (mg/d)	1291.7 ± 74.32	12	1166.7 ± 166.6	3	125	92.28	ns
Dietary omega 3 (mg/d)	538.8 ± 50.87	38	474.1 ± 83.0	9	64.73	32.13	ns
Vitamin E (mg/d)	14.28 ± 1.10	38	12.27 ± 1.80	9	2.00	0.70	ns
Vitamin B1 (mg/d)	1.54 ± 0.15	38	1.68 ± 0.30	9	0.14	0.15	ns
Vitamin B2 (mg/d)	2.07 ± 0.16	38	2.25 ± 0.35	9	0.18	0.19	ns
Vitamin B3 (mg/d)	23.4 ± 1.55	38	26.15 ± 3.01	9	2.78	1.46	ns
Vitamin B5 (mg/d)	3.31 ± 0.19	38	3.73 ± 0.35	9	0.43	0.16	ns
Vitamin B6 (mg/d)	1.20 ± 0.09	38	1.39 ± 0.26	9	0.19	0.17	ns
Vitamin B7 (mg/d)	33.88 ± 2.23	38	36.38 ± 4.92	9	2.49	2.69	ns
Iron (mg/d)	12.18 ± 0.76	38	13.07 ± 1.28	9	0.89	0.52	ns
Zinc (mg/d)	9.79 ± 0.60	38	10.31 ± 0.99	9	0.53	0.39	ns
Iodine (μg/d)	121.06 ± 7.64	38	118.53 ± 15.97	9	2.52	8.33	ns
Alcohol (g/d)	8.15 ± 1.67	27	1.29 ± 0.48	9	2.53	1.19	ns
Caffeine (mg/d)	538.5 ± 56.8	38	508.4 ± 143.8	9	30.06	87	ns

*Moderate exercise = 50–60% of maximum heart rate; Vigorous exercise = 70–85% of maximum heart rate; SE, standard error; ns, non-significant (p ≥ 0.05); M, male; F, female; mg/d, milligram per day; g/d, grams per day; μmol/L, micromole per liter; μg/d, microgram per day; pmol/L, picomole/liter. Bold values represent statistical significance at *p* < 0.05.*

The mean age of the participants in the adequate vitamin C group was lower than in those in the deficient group (Table 5). A majority of participants in the adequate group were female compared to a majority of males in the deficient group. A Chi-square likelihood ratio test revealed that there was no significant association between gender and vitamin C group (adequate/deficient) [χ^2^(1) = 2.80, *p* = 0.094]. Using the present data set, an interaction between gender by plasma vitamin C concentrations on cognitive function will be analyzed more extensively in a subsequent trial. There was an association between smoking and vitamin C group, with smokers more likely to display deficient vitamin C concentrations [χ^2^(1) = 7.23, *p* = 0.007]. Three smokers were present in the deficient group as opposed to 1 in the adequate group. Additionally, there was an association between taking prescribed medications and vitamin C group, with those not taking any prescribed medications more likely to display adequate vitamin C concentrations [χ^2^(1) = 5.75, *p* = 0.016].

A higher percentage (92.3%) of participants were on prescribed medications in the deficient group than in the group with adequate vitamin C levels (61.2%). No statistically significant differences were observed in mood between the two groups.

Serum vitamin B12 levels were significantly higher in the adequate than deficient group (*p* = 0.016). Additionally, no participants in the deficient group reported supplementation of either vitamin C, vitamin B12, or magnesium and only 1 reported vitamin D supplementation. Vitamin C daily dietary intake was not significantly higher in the adequate group compared to the deficient group (*p* ≥ 0.05). There were no other statistically significant differences in dietary intake between the other measured nutrients. Additional analyses revealed no significant differences in daily vitamin C consumption between those supplementing on vitamin C (*M* = 81.68 ± 24.98) and those not (*M* = 83.75 ± 19.20, *p* > 0.05) in the adequate vitamin C group. Within the adequate group, those supplementing on vitamin C exhibited significantly higher mean vitamin C concentrations (*M* = 66.65 ± 25.91) than those not supplementing (*M* = 40.59 ± 17.2, *p* < 0.01). Additional descriptive comparisons between the deficient and adequate groups in which vitamin C supplementers were excluded revealed similar results to when vitamin C supplementers were included (not shown).

### Cognitive Assessments

#### 3MS

The spread of 3MS scores was restricted, with scores grouping between 80 and 100. Spearman’s correlation analysis revealed no relationship between vitamin C concentrations and 3MS performance [*r*_s_(80) = 0.025, *p* = 0.82]. Additionally, an analysis of covariance (adjusting for age, vitamin B12, mg, vitamin C dose supplementation) revealed no significant differences in 3MS scores between the adequate and deficient group [*F*(1,78) = 2.6, *p* = 0.11].

#### HVLT-R and SDMT

**FIGURE 1:**
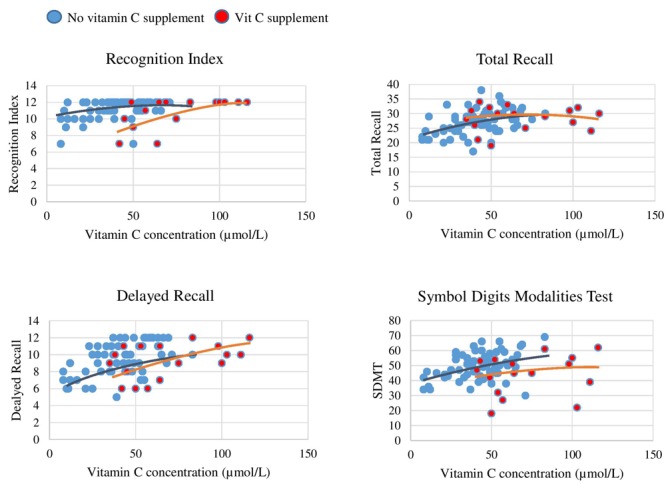
Plasma vitamin C concentrations and paper and pen test correlations. Red dots represent participants supplementing on vitamin C and blue dots those not supplementing on vitamin C. There is a plateau in performance on each of the cognitive measures once plasma concentration surpasses 70 μmol/L. In each graph there are two visual trend lines, one relating to data points of subgroup (a) participants not supplementing on vitamin C and subgroup (b) participants supplementing on vitamin C.

In order to assess the strength of association between plasma vitamin C and cognitive performance, mean plasma vitamin C concentrations were plotted against cognitive performance for each of the cognitive assessments in the HVLT-R and SDMT. Scatterplots were used to visualize cognitive performance between those supplementing on vitamin C relative to non-supplementers. Scatterplots are displayed in Figure 1.

As displayed by the scatterplots, there was a plateau in cognitive performance with increasing vitamin C concentrations (>70 μmol/L), representing a non-linear relationship. The plots further demonstrate that the participants who supplemented on vitamin C were those exhibiting this plateau. Vitamin C Supplementation time frame and dose did not vary greatly in this group. Spearman correlation analysis was conducted to investigate the strength of these non-linear relationships for each cognitive measure, as presented in Table 6.

**Table 6:** Correlation analyses for vitamin C concentrations and paper and pen assessments.

				Delayed	Recognition	Total
	Trial 1	Trial 2	Trial 3	recall	index	recall	SDMT
Spearman’s correlation *r*_s_(80)	0.13	0.46*	0.53*	0.43*	0.39*	0.42	0.26*
*p*-value	0.27	<**0**.**01**	<**0**.**01**	<**0**.**01**	<**0**.**01**	<**0**.**01**	**0**.**018**
Spearman’s correlation^#^ *r*_s_(60)	0.21	0.53*	0.56*	0.52*	0.42*	0.48*	0.47*
*p*-value	0.11	**0**.**01**	**0**.**01**	**0**.**01**	**0**.**01**	**0**.**01**	**0**.**01**

*^#^Non-vitamin C supplementers (n = 60), ^∗^significant (p < 0.05), r_s_(n) = Spearman correlation coefficient. Bold values represent statistical significance at *p* < 0.05.*

The Spearman correlations reveal a significant, positive relationship between vitamin C concentration and Trial 2, Trial 3, delayed recall, total recall and recognition index of the HVLT-R test (*p* < 0.01). There was a significant positive relationship between vitamin C concentrations and the SDMT test (*p* < 0.02).

When participants supplementing on vitamin C were not included in the correlational analysis, the relationship between vitamin C concentrations and each of the cognitive assessments appeared stronger (higher *r*_s_ value). Spearman correlations for these relationships are displayed in Table 6. The Spearman correlations (with vitamin C supplementers excluded) displayed significant positive correlations between vitamin C concentrations and the cognitive assessments on both the HVLT-R and the SDMT.

**FIGURE 2:**
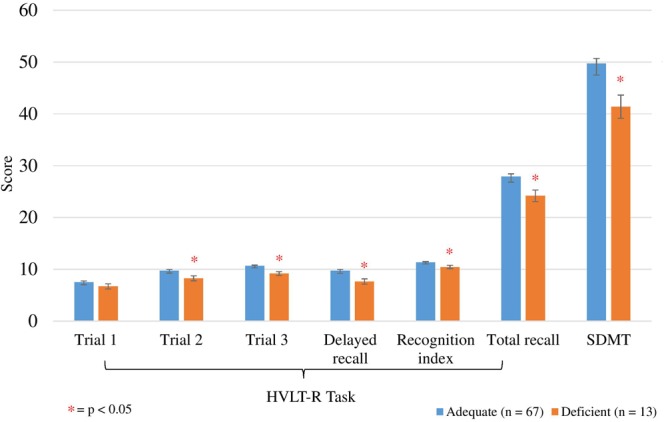
Comparison between adequate and deficient plasma vitamin C groups on HVLT-R cognitive measures and SDMT score. Participants grouped into adequate (*n* = 67) and deficient (*n* = 13) plasma vitamin C groups. Comparison assessed with an ANCOVA analysis, controlling for vitamin C supplement dose (Trials 1, 2, 3, Total recall), age (SDMT) and number of prescription medications (recognition index). Trials 1, 2, 3, delayed recall/recognition index scored out of 12 points; total recall scored out of 36 points. HVLT-R, Hopkins Verbal Learning Test Revised; SDMT, Symbol Digits Modalities Test; ^∗^ significant (*p* < 0.05). Delayed recall/recognition index scored out of 12 points; total recall scored out of 36 points.

The mean scores of the HVLT-R cognitive assessments and SDMT for the vitamin C adequate and deficient groups are displayed in Figure 2. ANCOVA analyses were performed to compare performance on the HVLT-R cognitive assessments and SDMT between adequate and deficient vitamin C concentrations while controlling for potential covariates (Table 7). Vitamin C supplementation dose was a significant covariate for four HVLT-R measures, number of prescribed medications for the recognition index measure and age for the SDMT test.

**Table 7:** Comparison between adequate and deficient plasma vitamin C groups on HVLT-R cognitive measures and SDMT score.

Paper and pen cognitive assessment	Mean score ±*SE* (*n* = 80)		Covariates	Parameter estimates			Differences between adequate vs. deficient vitamin C level groups		
All-vitamin C supplementers included	Adequate (*n* = 67)	Deficient (*n* = 13)		*B*	*SE*	*p*-value	*M*	*SE*	*p*-value
*HVLT-R*	
Trial 1	7.52 ± 0.21	6.69 ± 0.48	None				0.83	0.52	0.11
Trial 2	9.76 ± 0.21	8.25 ± 0.49	Vit C supplement dose	0.001	0	0.05	1.51	0.53	0.006*
Trial 3	10.64 ± 0.16	9.17 ± 0.37	Vit C supplement dose	0.001	0	**0.011**	1.46	0.40	0.001*
Delayed recall	9.74 ± 0.22	7.64 ± 0.51	Vit C supplement dose	0.001	0	0.06	2.10	0.56	<0.001*
Total recall	27.93 ± 0.48	24.19 ± 1.11	Vit C supplement dose	0.002	0.001	**0.045**	3.75	1.21	0.003*
Recognition index	11.37 ± 0.15	10.35 ± 0.33	Number of meds	–0.20	0.082	**0.018**	1.02	0.36	0.007*
SDMT	49.73 ± 0.98	41.38 ± 2.25	Age	–0.36	0.058	<**0.001**	5.06	2.47	0.044*
All except vitamin C supplementers	Adequate (*n* = 47)	Deficient (*n* = 13)							
Trial 1	7.60 ± 0.25	6.69 ± 0.48	None				0.90	0.54	0.10
Trial 2	9.72 ± 0.24	8.08 ± 0.45	None				1.65	0.50	0.002*
Trial 3	10.47 ± 0.20	9.00 ± 0.38	None				1.47	0.44	0.001*
Delayed recall	9.81 ± 0.26	7.46 ± 0.49	None				2.35	0.55	<0.001*
Total recall	27.85 ± 0.59	23.77 ± 1.13	None				4.08	1.28	0.002*
Recognition index	11.48 ± 0.15	10.34 ± 0.3	None				1.14	0.34	0.002*
SDMT	51.24 ± 1.0	44.06 ± 1.98	Age	–0.19	0.068	**0.006**	7.18	2.27	0.003*
			Years of education	0.011	0.006	0.07			
			Vit B12 supplement dose	1.34	0.76	0.08			

*^∗^p < 0.05; SE, standard error of the mean; Vit, vitamin C. Bold values represent statistical significance at *p* < 0.05.*

Significantly higher scores were observed on trials 2 and 3, delayed and total recall, and recognition on the HVLT-R in the adequate vitamin C group (Figure 2 and Table 7). On average, the adequate vitamin C group recalled close to 2 words more on both trials 2 and 3, delayed recall and recognized 1 more word based on the recognition index. Additionally, on average, those in the adequate vitamin C group recalled close to 4 words more on total recall. This group also had significantly higher SDMT scores then the deficient group (Table 7), with an average higher score of 7 points (1 SD).

An additional ANCOVA analysis was conducted (Table 7) comparing the adequate and deficient vitamin C groups with the exclusion of vitamin C supplementers (Table 7). As Table 7 demonstrates, when vitamin C supplementers were excluded, the cognitive differences between the adequate and deficient vitamin C groups on the HVLT-R task and SDMT did not vary greatly in comparison to when the supplementers were included. ANCOVA analyses were conducted in which each of the HVLT-R cognitive assessments and SDMT scores were compared between those supplementing on vitamin C (*n* = 20) and non-supplementers (*n* = 47) in the adequate group. Mean values on these cognitive tasks between those supplementing on vitamin C and non-vitamin C supplementers in the adequate group were not significantly different (Supplementary Table S1).

#### SUCCAB

Mean vitamin C plasma concentrations were correlated with the reaction times for each of the SUCCAB tasks (Table 8). Lower reaction times were indicative of quicker response times.

**Table 8:** Correlational analysis between vitamin C concentrations and SUCCAB task reaction times.

	Simple	Choice	Immediate			Spatial		Delayed
	reaction	reaction	recognition	Congruent	Incongruent	working	Contextual	recognition
	time	time	memory	Stroop	Stroop	memory	memory	memory
Spearman correlation *r*_s_(80)	–0.04	0.09	–0.17	–0.38*	–0.23	–0.24*	–0.15	–0.27*
*p*-value	0.72	0.45	0.14	0.01	**0.05**	**0.03**	0.18	**0.016**
Spearman correlation^#^ *r*_s_(60)	–0.11	–0.29*	–0.21	–0.51*	–0.40*	–0.26*	–0.29*	–0.34*
*p*-value	0.39	**0.025**	0.11	**0.01**	**0.002**	**0.04**	**0.027**	**0.009**

*^#^Non-vitamin C supplementers (n = 60), ^∗^significant (p < 0.05), r_*s*_(n) = Spearman correlation coefficient. Bold values represent statistical significance at *p* < 0.05.*

The Spearman correlation (*n* = 80) revealed an inverse, significant, negative relationship between mean vitamin C plasma concentrations and reaction time on the congruent Stroop, spatial working memory and delayed recognition memory tasks. Similar to the paper and pen assessments, we conducted subgroup correlational analysis excluding participants supplementing on vitamin C, and found that the relationship between vitamin C concentrations and reaction times for each of the tasks appeared stronger (higher *r*_s_ values) than when vitamin C supplementers were included.

The Spearman correlations (with vitamin C supplementers excluded) displayed statistically significant, inverse correlations between vitamin C concentrations and reaction time on choice reaction time, spatial working memory, the congruent and incongruent Stroop tasks and contextual memory tasks and a significant correlation on delayed recognition memory (Table 8, *n* = 60). The correlations between vitamin C and choice reaction time, incongruent Stroop and contextual memory were not significant when vitamin C supplementers were included (*n* = 80).

**FIGURE 3:**
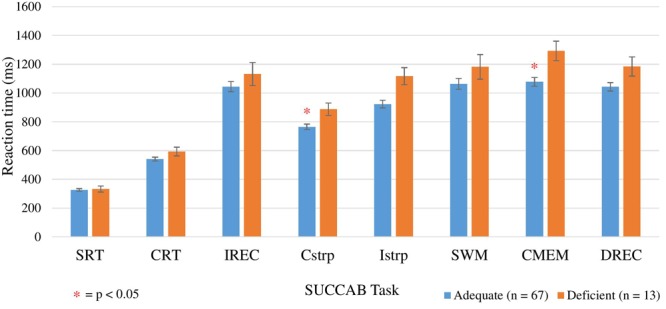
Comparison between adequate and deficient plasma vitamin C groups on SUCCAB reaction time. Participants grouped into adequate (*n* = 67) and deficient (*n* = 13) plasma vitamin C groups. Comparison assessed with an ANCOVA analysis, controlling for age on congruent and incongruent Stroop, numbers of medications and years of education on contextual memory and number of medications on delayed recognition memory. Reaction time assessed in milliseconds. SUCCAB, Swinburne University Computerized Cognitive Assessment Battery; SRT, simple reaction time; CRT, choice reaction time; IREC, immediate recognition memory; Cstrp, congruent Stroop; Istrp, incongruent Stroop; SWM, spatial working memory; CMEM, contextual memory; DREC, delayed recognition memory; ^∗^ significant (*p* < 0.05).

Spearman correlation ANCOVA analyses revealed age to be a significant covariate for the reaction times on the congruent and incongruent Stroop tasks, number of prescribed medications for reaction time on the contextual memory and delayed recognition tasks, and years of education for reaction time on the contextual memory task (Table 9). Analyses revealed lower (faster) mean reaction times in the adequate vitamin C group compared to the deficient for all of the SUCCAB tasks. Significant differences between the groups were observed on the congruent Stroop and contextual memory tasks (Figure 3). An additional ANCOVA analysis was conducted (Table 9) comparing the adequate and deficient vitamin C groups with the exclusion of vitamin C supplementers (Table 9).

**Table 9:** Comparison between adequate and deficient plasma vitamin C groups on SUCCAB reaction time.

SUCCAB Task (reaction time- ms)	Mean score ±*SE* (*n* = 80)		Covariates	Parameter estimates			Differences between adequate vs. deficient vitamin C level groups		
All-vitamin C supplementers included	Adequate (*n* = 67)	Deficient (*n* = 13)		*B*	*SE*	*p*-value	Mean	*SE*	*p*-value
Simple reaction time	325.88 ± 9.43	332.76 ± 21.46	None				6.87	23.39	0.78
Choice reaction time	541.14 ± 13.53	593.09 ± 30.75	None				51.95	33.35	0.13
Immediate recognition memory	1044.40 ± 34.93	1131.92 ± 80.53	None				87.52	88.33	0.35
Congruent Stroop	764.84 ± 18.73	886.79 ± 43.19	Age	2.66	1.11	0.02	121.94	47.37	0.012*
Incongruent Stroop	923.07 ± 26.47	1117.30 ± 59.35	Age	2.92	1.57	0.07	94.23	65.50	0.16
Spatial working memory	1063.56 ± 37.75	1181.19 ± 85.70	None				117.63	93.65	0.21
Contextual memory	1077.56 ± 30.01	1293.04 ± 67.86	Number of meds	48.73	18.83	0.012	215.47	74.51	0.005*
			Years of education	37.58	17.13	0.031			
Delayed recognition memory	1043.02 ± 29.38	1184.69 ± 66.77	Number of meds	36.92	16.57	0.029	141.67	73.20	0.057
**All except vitamin C supplementers**	Adequate (*n* = 47)	Deficient (*n* = 13)							
Simple reaction time	325.72 ± 12.10	332.76 ± 23.00	None				7.04	25.99	0.79
Choice reaction time	540.05 ± 17.97	593.09 ± 33.14	None				53.05	38.58	0.17
Immediate recognition memory	1009.50 ± 38.66	1139.69 ± 73.51	None				130.19	83.06	0.12
Congruent Stroop	757.50 ± 24.09	879.39 ± 87.41	Age	2.78	1.45	0.06	121.89	54.25	0.03*
Incongruent Stroop	901.58 ± 33.49	1040.99 ± 62.31	None				139.40	70.74	0.054
Spatial working memory	1095.58 ± 43.77	1089.29 ± 87.01	Number of meds	83.50	27.36	0.003	6.29	99.89	0.95
Contextual memory	1038.39 ± 35.46	1305.82 ± 66.71	None				266.92	75.55	0.001*
Delayed recognition memory	1020.65 ± 33.52	1206.30 ± 63.06	None				185.65	71.41	0.012*

*^∗^p < 0.05; SE, standard error of the mean; ms, milliseconds*.

As Table 9 demonstrates, when vitamin C supplementers were excluded, significant differences between the groups were also observed on the congruent Stroop, contextual memory and additionally, the delayed recognition memory task. Mean reaction times on each SUCCAB task between those supplementing on vitamin C and non-vitamin C supplementers in the adequate group were not significantly different (Supplementary Table S2).

Additionally, analyses were conducted on the overall ratio performance (accuracy/reaction time) of each SUCCAB task. Mean vitamin C plasma concentrations were correlated with the mean ratio of each SUCCAB task (Table 10). The Spearman correlations revealed a positive, significant relationship between mean vitamin C plasma concentrations and ratios on the congruent Stroop and spatial working memory.

**Table 10:** Correlational analysis between vitamin C concentrations and SUCCAB ratios.

	Simple	Choice	Immediate			Spatial		Delayed
	reaction	reaction	recognition	Congruent	Incongruent	working	Contextual	recognition
	time	time	memory	Stroop	Stroop	memory	memory	memory
Spearman’s correlation *r*_s_(80)	0.04	0.09	0.16	0.38*	0.04	0.25*	0.10	0.21
*p*-value	0.72	0.44	0.16	<0.01	0.72	**0.02**	0.37	0.07
Spearman’s correlation^#^ *r*_s_(60)	0.11	0.26	0.24	0.52*	0.14	0.37*	0.29*	0.28*
–value	0.39	0.26	0.07	<**0.01**	0.29	<**0.01**	**0.026**	**0.03**

*^#^Non-vitamin C supplementers (n = 60), ^∗^significant (p < 0.05), r_s_(n) = Spearman correlation coefficient. Bold values represent statistical significance at *p* < 0.05.*

The Spearman correlation (with vitamin C supplementers excluded) displayed statistically significant, correlations between vitamin C concentrations and performance ratios on the congruent Stroop, spatial working memory and contextual memory, and additionally contextual memory and delayed recognition memory (Table 10, *n* = 60).

**FIGURE 4:**
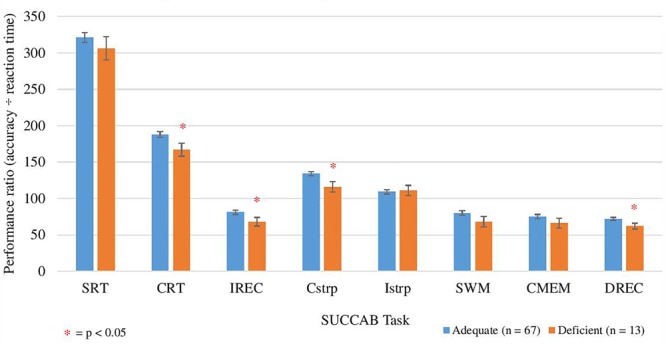
Comparison between adequate and deficient plasma vitamin C groups on SUCCAB ratio performance. Participants grouped into adequate (*n* = 67) and deficient (*n* = 13) plasma vitamin C groups. Comparison assessed with an ANCOVA analysis, controlling for age on congruent and incongruent Stroop, numbers of medications and years of education on contextual memory and number of medications on delayed recognition memory. Reaction time assessed in seconds. SUCCAB, Swinburne University Computerized Cognitive Assessment Battery; SRT, simple reaction time; CRT, choice reaction time; IREC, immediate recognition memory; Cstrp, congruent Stroop; Istrp, incongruent Stroop; SWM, spatial working memory; CMEM, contextual memory; DREC, delayed recognition memory; ^∗^ significant (*p* < 0.05).

ANCOVA analyses revealed age to be a significant covariate for the ratios on the congruent and incongruent Stroop, spatial working memory and contextual memory. Analyses revealed higher ratios for each of the SUCCAB tasks in the adequate vitamin C group in comparison to the deficient group. Significant differences between the groups were observed on immediate recognition memory, congruent Stroop, choice reaction time and delayed recognition memory (Table 11 and Figure 4). As Table 11 demonstrates, when vitamin C supplementers were excluded, the mean scores on each of the cognitive measures on the HVLT-R task and SDMT did not vary greatly in comparison to when the supplementers were included.

**Table 11:** Comparison between adequate and deficient plasma vitamin C groups on SUCCAB task ratio.

SUCCAB Task (ratio)	Mean score ±*SE* (*n* = 80)		Covariates	Parameter estimates			Differences between adequate vs. deficient vitamin C level groups		
All-vitamin C supplementers included	Adequate (*n* = 67)	Deficient (*n* = 13)		*B*	*SE*	*p*-value	Mean	*SE*	*p*-value
Simple reaction time	320.59 ± 6.98	305.81 ± 16.09	None				14.77	17.14	0.39
Choice reaction time	187.53 ± 3.90	167.23 ± 8.86	None				20.29	9.98	0.039*
Immediate recognition memory	81.47 ± 2.52	67.96 ± 5.72	None				13.77	6.26	0.031*
Congruent Stroop	133.69 ± 2.97	116.46 ± 6.81	Age	–0.486	0.177	**0.008**	17.24	7.48	0.024*
Incongruent Stroop	109.137 ± 2.97	109.94 ± 6.68	Age	–0.523	0.176	**0.004**	0.79	7.34	0.91
Spatial working memory	80.27 ± 2.95	68.02 ± 6.81	Age	–0.519	0.176	**0.004**	12.25	7.47	0.105
Contextual memory	75.23 ± 3.24	65.24 ± 7.36	Age	–0.350	1.910	0.07	9.98	8.10	0.22
Delayed recognition memory	71.91 ± 1.93	62.41 ± 4.35	None				9.50	4.76	0.049*
**All except vitamin C supplementers**	Adequate (*n* = 47)	Deficient (*n* = 13)							
Simple reaction time	322.93 ± 8.56	305.82 ± 10.28	None				17.17	18.39	0.356
Choice reaction time	189.28 ± 5.077	167.24 ± 9.65	None				22.05	10.91	0.048*
Immediate recognition memory	83.96 ± 3.84	68.0 ± 5.74	None				16.26	6.48	0.015*
Congruent Stroop	137.24 ± 3.84	112.68 ± 7.22	None				24.56	8.18	0.004*
Incongruent Stroop	110.02 ± 3.80	111.57 ± 7.34	Age	–0.580	0.23	**0.012**	1.55	8.44	0.86
Spatial working memory	80.69 ± 3.44	70.09 ± 6.76	Age	–0.605	0.21	0.005	10.61	7.74	0.176
Contextual memory	80.45 ± 3.96	62.55 ± 7.44	None				17.91	8.43	0.038*
Delayed recognition memory	73.01 ± 3.0	62.42 ± 4.33	None				10.60	4.90	0.035*

*^∗^p < 0.05; SE, standard error of the mean. Bold values represent statistical significance at *p* < 0.05.*

An additional ANCOVA analysis was conducted in which each of the HVLT-R cognitive assessments and SDMT scores were compared between those supplementing on vitamin C (*n* = 20) and non-supplementers (*n* = 47) in the adequate group. Mean performance ratio on only the contextual memory task was significantly higher in the adequate vitamin C group not supplementing on vitamin C than in those self-reporting vitamin C supplementation (Supplementary Table S3).

Finally, results revealed no significant correlations between the HVLT-R scores and any of the reaction times and ratios on the SUCCAB tasks. Significant correlations were observed between the SDMT scores and ratios on immediate recognition memory, congruent and incongruent Stroop, spatial working memory and contextual memory.

## Discussion

Correlational analyses revealed a positive, significant correlation between vitamin C concentrations and performance ratio (accuracy ÷ reaction time) on the congruent Stroop, and spatial working memory tasks on the sensitive computerized SUCCAB. A significantly higher covariate adjusted performance ratio was only demonstrated on the choice reaction time, immediate recognition memory, congruent Stroop, and delayed recognition tasks in the group with adequate vitamin C levels.

Significantly higher covariate adjusted scores in immediate recall, delayed recall, total recall on the HVLT-R and SDMT scores were observed in those participants with adequate plasma vitamin C concentrations. A plateau in cognitive performance was observed on tasks in those supplementing with vitamin C and displaying vitamin C concentrations exceeding 70 μmol/L. ANCOVA analyses also indicated no additional cognitive benefits in those who self-reported vitamin C supplementation.

This is the first study to date to examine the link between vitamin C concentrations and cognition with the use of the highly sensitive computerized test SUCCAB, which can detect millisecond differences in reaction time (Pipingas et al., 2010), whereby 100 ms difference is related to 10 years of age on a the most difficult task (Pipingas et al., 2010). However, 8 studies (La Rue et al., 1997; Perrig et al., 1997; Lindeman et al., 2000; Grodstein et al., 2003; Péneau et al., 2011; Devore et al., 2013; Beydoun et al., 2015; Nooyens et al., 2015) have administered cognitive tests assessing cognitive domains that are similar to the SUCCAB tasks such as reaction time, visual perception, executive function, immediate and long term recognition/recall (tests include delayed word recall, trail making forward/backward test, Benton visual retention test, Halstead-Reitan categories test, etc.). These studies generally found a positive association between plasma vitamin C and cognition.

The positive relationship demonstrated between vitamin C concentrations and delayed recall, total recall and recognition on the HVLT-R assessment are consistent with one previous cross-sectional study using a computerized cognitive test (assessed working, implicit and explicit memory) (Perrig et al., 1997; Péneau et al., 2011) but contrary to a number of studies (Peacock et al., 2000; Grodstein et al., 2003) that used a lower quality design.

The positive relationship between vitamin C concentrations and SDMT scores found higher scores in those with adequate levels is consistent with a cross-sectional study (Sato et al., 2006) in which the highest fifth of plasma vitamin C concentrations were associated with better symbol digit substitution scores. No such association was discovered in a study which also used the letter substitution task but with a lower quality design by using a food frequency assessment instead of plasma to assess vitamin C concentration (Nooyens et al., 2015).

Vitamin C concentrations displayed an inverse relationship with reaction time on contextual memory with a trade-off on accuracy in the group in the adequate vitamin C level group. The congruent Stroop task performance was moderately related to plasma vitamin C concentrations, with a significantly higher performance and faster reaction time in the adequate vitamin C group. This finding is in contrast to a previous prospective study that used a FFQ to estimate vitamin C concentrations (Nooyens et al., 2015), but consistent with a higher quality randomized controlled trial using a multivitamin (80 mg vitamin C) (Chandra, 2001).

The choice reaction time performance ratio was positively associated with plasma vitamin C concentrations, with a significantly higher performance ratio in the adequate vitamin C group. These findings are in line with a previous cross-sectional (Perrig et al., 1997) and a 6-year prospective study (La Rue et al., 1997) which assessed visuo-spatial performance.

The significant correlations between the SDMT and a number of SUCCAB task ratios can be explained by reaction time and processing speed being the key components tested in both tasks. Both the SUCCAB and SDMT test fluid intelligence, a component of intelligence which relates to reasoning and solving novel problems, independent of any knowledge from the past. This intelligence peaks in young adulthood and then steadily declines. The cognitive decline can be attributed to lack of practice, along with age-related changes in the brain may contribute to the decline (Cunningham et al., 1975).

Lack of correlation between the HVLT-R measures and SUCCAB task performance may be explained by the HVLT-R not assessing reaction time and speed but rather focusing on accuracy of word recall and recognition, testing components of both fluid and crystalized intelligence. The SUCCAB tasks are based on visual processing whereas the HVLT-R is relying on verbal and auditory processing through spoken words. Previous research has demonstrated an asymmetry between auditory and visual processing (Cohen et al., 2009) and a stability of crystallized intelligence across most of adulthood which begins to decline after the age of 65 (Cunningham et al., 1975).

A number of explanations/mechanisms may attribute the observed results on both the paper and pen assessments and the computerized tests. It is established that vitamin C in higher concentrations stimulates the production of acetylcholine (Torda and Wolff, 1945) and an increase in brain acetylcholine receptor (AcChoR) numbers and distribution by influencing AcChoR expression through mRNA (Knaack and Podleski, 1985; Knaack et al., 1986). Vitamin C is involved as a co-factor in the conversion of dopamine into norepinephrine within the brain and adrenals (Harrison et al., 2009). Both norepinephrine and acetylcholine have been shown to play vital roles attention, focus and memory (Klinkenberg et al., 2011). Additionally, results may be explained by the involvement of vitamin C in serotonin production and neuronal absorption (Gupta et al., 2014), energy production through the synthesis of L-carnitine (Rebouche, 1991) and ROS scavenger action, enabling optimal mitochondrial function (Harrison and May, 2009).

The plateau in cognitive function observed in our study in those supplementing with vitamin C and displaying plasma concentrations >70 μmol/L can be explained by the uptake and maintenance of vitamin C in the CNS. Based on previous research, it has been established that in neuronal cells, the apparent Michaelis–Menten transport kinetics (Km) for ascorbate appears to be somewhat high (113 μmol/L); this affinity corresponds to plasma vitamin C concentrations of 30–60 μmol/L (May et al., 2006).

Although higher plasma ascorbic acid concentrations generally result in higher CSF concentrations (CSF: plasma ratio of about 3–4:1) (Quinn et al., 2003), these concentrations start to reach a steady state as plasma concentrations surpass 60 μmol/L. As plasma concentrations decline, relatively more ascorbate is pumped into the CSF in order to maintain homeostasis (May et al., 2006; Bowman et al., 2009). The variability in CSF starts to become apparent when the duration of vitamin C deficiency is extended. Thus, plasma vitamin C relates brain vitamin C status in a narrow window (<30 μmol/L) (Travica et al., 2017).

The cognitive plateau may be further explained by a possible over stimulation of norepinephrine in those displaying higher plasma vitamin C levels. Studies have indicated that norepinephrine does not display linear cognitive effects; instead, its modulation of cognitive and neuronal function maps on an Inverted-U curve (Arnsten and Li, 2005). During hyper noradrenergic states, noradrenergic α1 and β receptors are activated, leading to reduced neuronal signal efficiency and possible impairment of attentional selectivity and locomotor hyperactivity and distractibility (Arnsten and Li, 2005).

A strength of our study is its incorporation of two biochemical techniques in determining plasma vitamin C concentrations, due to the unstable nature of vitamin C in plasma. Furthermore, the present study is the first to assess vitamin C concentrations in a broad spectrum of cognitive abilities in a cognitively intact sample. The lack of spread in scores on the 3MS screening test is consistent with a number of previous cross-sectional studies (Jama et al., 1996; Ortega et al., 1997) and those discussed in a recent systematic review (Travica et al., 2017). Although the 3MS scores revealed minor variability, the other cognitive assessments were capable of detecting subtle, significant differences. Given the extensive link between vitamin B12 and cognition, a further strength of the present study is the use of serum vitamin B12 as a potential covariate.

The comparison between the food frequency questionnaire and biochemical analyses highlighted a moderate strength, significant correlation between the vitamin C measured FFQ intakes and the biochemical analyses, highlighting possible assessment inadequacies, with the lower quality study designs in previous studies. Limitations with the use of food frequency questionnaires have been well established, ranging from recall errors/bias (Pearson et al., 2017), nutrient content alterations as a result of storage and cooking (Weinstein et al., 2001), and no insight into nutrient absorption (Harrison, 2012).

Given the inadequacy of using a FFQ, our study was limited to only assessing the consumption of 20 nutrients. Other substances which have been shown to influence cognitive function such as choline (Poly et al., 2011), flavonoids (Letenneur et al., 2007) and plant based polyphenols (Vauzour, 2012), were not studied here. Although mean plasma vitamin C concentrations were higher in those supplementing on vitamin C in our study, dose and frequency were assessed using a self-report method, with no monitoring of when during the day and for how long (over 1 week prior to testing) vitamin C supplements were taken. Clinical research examining the pharmacokinetics of oral vitamin C intake has demonstrated that plasma vitamin C concentrations may exceed 100 μmol/l in healthy participants taking oral vitamin C doses over 200 mg, and that these levels can remain at these concentrations for as long as 24 h post-supplementation (depending on health/oxidative stress, etc.) (Padayatty et al., 2004). The participants displaying levels over 100 μmol/l within our sample were only those who reported vitamin C supplementation. All testing sessions were conducted in the morning with participants fasting. The individuals who self-reported vitamin C supplementation may have supplemented within 12–24 h prior to testing and reached plasma concentrations exceeding 100 μmol/l when blood was taken. Furthermore, those attaining adequate plasma vitamin C concentrations from diet performed similarly on the cognitive assessments to those supplementing on vitamin C.

There are a number of limitations arising in this study. The cross-sectional design is not suggestive of a causal relationship. Plasma vitamin C levels are dependent on recent dietary intake, therefore they are not very reproducible and also not representative of long-term consumption, which is possibly more relevant to neurologic outcome than short-term consumption (Bowman et al., 2009). The duration of depleted vitamin C concentrations were not known in our sample. This finding would be crucial, as previous studies (Spector and Lorenzo, 1973; Hornig, 1975) have demonstrated that the duration of deficiency seems to affect CNS concentrations to a greater extent than the amount of depletion.

Although our study used the Bond–Lader mood questionnaire before the cognitive assessments, we did not assess mood states post cognitive assessment. This final mood assessment would have provided an insight into the emotional affects that the cognitive tasks were having on participants by assessing subjective variables such as stress, fatigue, and energy.

An important area to be considered in future studies is cardiovascular health. Given the brain’s reliability on efficient cerebral blood flow for the delivery of vital biochemical, such as oxygen and glucose, previous studies have systematically demonstrated an effect of blood pressure and arterial stiffness on cognition (Fujishima et al., 1995; Tsao et al., 2013; Singer et al., 2014). Given the large percentage of participants in our study on blood pressure medications, cardiovascular health may have played a role in cognition over and above vitamin C concentrations.

Furthermore, additional biomarkers (other than Vitamin B12) which can influence both vitamin C concentrations and cognition could also be considered as potential covariates. These include corticosteroids such as cortisol (Sindi et al., 2012), inflammatory markers such as c-reactive protein alongside cytokines (McAfoose and Baune, 2009), white blood cells such as lymphocytes and neutrophils (Acid, 1977) and biomarkers reflecting oxidative stress such as glutathione peroxidase and glutathione (Revel et al., 2015). While our study measured plasma vitamin C and vitamin B12 levels biochemically, additional nutrients important for cognitive health such as folate, vitamin B6, vitamin D, and homocysteine should be assessed by blood tests. Alongside cardiovascular health markers, blood biomarkers which have consistently demonstrated links with both cognition and vitamin C concentrations such as cortisol, inflammatory markers and white blood cells should be considered. Although the present study did find a link between vitamin C levels and a variety of cognitive domains, future studies could confirm aspects of our results by focusing on specific cognitive domains, such as spatial working memory, and develop hypotheses and use sample sizes based on these specific cognitive domains.

## Conclusion

In summary, using vitamin C plasma levels and validated pen and paper cognitive tests and age-sensitive computerized cognitive tests suitable for comparative analysis in cognitively intact participants, we found a significant association between vitamin C concentrations and performance on tasks involving attention, focus, working memory, decision speed, both delayed and total recall, and recognition.

In line with our hypothesis and biological roles of vitamin C on the central nervous system, significantly higher cognitive scores in adults with adequate plasma vitamin C levels (≥28 μmol/L) were predominantly observed on tasks involving decision speed and inhibition, focus, attention, recall (both immediate and delayed) and recognition compared to those with deficient vitamin C levels (<28 μmol/L). A plateau in cognitive performance was observed on tasks involving attention, focus, immediate and delayed recall in those supplementing with vitamin C and displaying vitamin C concentrations exceeding 70 μmol/L, a finding consistent with the homeostatic mechanism of vitamin C in the central nervous system. This is an indication of no additional cognitive benefits in those who self-reported vitamin C supplementation among those with non-deficient levels of plasma vitamin C. The findings from this cross-sectional study warrant future cohort or longitudinal, randomized controlled trials. This would not only enable repeated measures of vitamin C and cognition but also the assessment of a causal relationship between vitamin C and cognition.

## Data Availability

All datasets generated for this study are included in the manuscript and/or the Supplementary Files.

## Author Contributions

AvS and NT conceptualized the study in discussion with KR and AP. IH provided software and statistical expertise. NT undertook data analysis and interpreted findings in discussion with KR and IH. NT prepared the manuscript with contributions from all co-authors (KR, AP, AvS, IH, and AnS). All authors approved the final version.

## Conflict of Interest Statement

AnS and AP have received research funding, consultancy, travel support, and speaking fees from the nutrition and supplement industry. The remaining authors declare that the research was conducted in the absence of any commercial or financial relationships that could be construed as a potential conflict of interest.

## References

[B1] Acid A. (1977). The effect of ascorbic acid supplementation on some parameters of the human immunological defence system. *Int. J. Vitam. Nutr. Res.* 47 248–257 914459

[B2] Arnsten A. F. Li B. -M. (2005). Neurobiology of executive functions: catecholamine influences on prefrontal cortical functions. *Biol. Psychiatry* 57 1377–1384. 10.1016/j.biopsych.2004.08.019 15950011

[B3] Basu T. Donaldson D. (2003). “Scurvy” in *Encyclopedia of Food Sciences and Nutrition* eds Caballero B. Finglas P. Toldra F. (Cambridge, MA: Academic Press

[B4] Beydoun M. A. Kuczmarski M. F. Kitner-Triolo M. H. Beydoun H. A. Kaufman J. S. Mason M. A. (2015). Dietary antioxidant intake and its association with cognitive function in an ethnically diverse sample of US adults. *Psychosom. Med.* 77 68–82. 10.1097/PSY.0000000000000129 25478706 PMC4597309

[B5] Bond A. Lader M. (1974). The use of analogue scales in rating subjective feelings. *Br. J. Med. Psychol.* 47 211–218. 10.1111/j.2044-8341.1974.tb02285.x

[B6] Bowman G. L. Dodge H. Frei B. Calabrese C. Oken B. S. Kaye J. A. (2009). Ascorbic acid and rates of cognitive decline in Alzheimer’s disease. *J. Alzheimers Dis.* 16 93–98. 10.3233/JAD-2009-0923 19158425 PMC2674290

[B7] Brandt J. (1991). The hopkins verbal learning test: development of a new memory test with six equivalent forms. *Clin. Neuropsychol.* 5 125–142. 10.1080/13854049108403297

[B8] Brubacher D. Moser U. Jordan P. (2000). Vitamin C concentrations in plasma as a function of intake: a meta-analysis. *Int. J. Vitam. Nutr. Res.* 70 226–237. 10.1024/0300-9831.70.5.226 11068703

[B9] Carr A. C. Frei B. (1999). Toward a new recommended dietary allowance for vitamin C based on antioxidant and health effects in humans–. *Am. J. Clin. Nutr.* 69 1086–1107. 10.1093/ajcn/69.6.1086 10357726

[B10] Castro M. A. Angulo C. Brauchi S. Nualart F. Concha I. I. (2008). Ascorbic acid participates in a general mechanism for concerted glucose transport inhibition and lactate transport stimulation. *Pflügers Archi.* 457 519–528. 10.1007/s00424-008-0526-1 18506475

[B11] Chandra R. K. (2001). RETRACTED: effect of vitamin and trace-element supplementation on cognitive function in elderly subjects. *Nutrition* 17 709–712. 10.1016/S0899-9007(01)00610-4 11527656

[B12] Chen J. Berry M. J. (2003). Selenium and selenoproteins in the brain and brain diseases. *J. Neurochem.* 86 1–12. 10.1046/j.1471-4159.2003.01854.x 12807419

[B13] Chung W. Y. Chung J. K. O. Szeto Y. T. Tomlinson B. Benzie I. F. (2001). Plasma ascorbic acid: measurement, stability and clinical utility revisited. *Clin. Biochem.* 34 623–627. 10.1016/S0009-9120(01)00270-3 11849621

[B14] Cohen M. A. Horowitz T. S. Wolfe J. M. (2009). Auditory recognition memory is inferior to visual recognition memory. *Proc. Natl. Acad. Sci. U.S.A.* 106 6008–6010. 10.1073/pnas.0811884106 19307569 PMC2667065

[B15] Covarrubias-Pinto A. Acuña A. I. Beltrán F. A. Torres-Díaz L. Castro M. A. (2015). Old things new view: ascorbic acid protects the brain in neurodegenerative disorders. *Int. J. Mol. Sci.* 16 28194–28217. 10.3390/ijms161226095 26633354 PMC4691042

[B16] Cunningham W. R. Clayton V. Overton W. (1975). Fluid and crystallized intelligence in young adulthood and old age. *J. Gerontol.* 30 53–55. 10.1093/geronj/30.1.53 1109393

[B17] De Benoist B. McLean E. Andersson M. Rogers L. (2008). Iodine deficiency in 2007: global progress since 2003. *Food Nutr. Bull.* 29 195–202. 10.1177/156482650802900305 18947032

[B18] Deicher R. Hörl W. H. (2003). Vitamin C in chronic kidney disease and hemodialysis patients. *Kidney Blood Press. Res.* 26 100–106. 10.1159/000070991 12771534

[B19] Devore E. E. Kang J. H. Stampfer M. J. Grodstein F. (2013). The association of antioxidants and cognition in the Nurses’ health study. *Am. J. Epidemiol.* 177 33–41. 10.1289/EHP1691 23221724 PMC3590037

[B20] Eldridge C. F. Bunge M. B. Bunge R. P. Wood P. M. (1987). Differentiation of axon-related Schwann cells in vitro. I. Ascorbic acid regulates basal lamina assembly and myelin formation. *J. Cell Biol.* 105 1023–1034. 10.1083/jcb.105.2.1023 3624305 PMC2114758

[B21] Figueroa-Méndez R. Rivas-Arancibia S. (2015). Vitamin C in health and disease: its role in the metabolism of cells and redox state in the brain. *Front. Physiol.* 6:397. 10.3389/fphys.2015.00397 26779027 PMC4688356

[B22] Forstmann B. U. Tittgemeyer M. Wagenmakers E. -J. Derrfuss J. Imperati D. Brown S. (2011). The speed-accuracy tradeoff in the elderly brain: a structural model-based approach. *J. Neurosci.* 31 17242–17249. 10.1523/JNEUROSCI.0309-11.2011 22114290 PMC6623864

[B23] Fox H. M. Linkswiler H. (1961). Pantothenic acid excretion on three levels of intake. *J. Nutr.* 75 451–454. 10.1093/jn/75.4.451 13894371

[B24] Frei B. Traber M. G. (2001). The new US Dietary Reference Intakes for vitamins C and E. *Redox Rep.* 6 5–9. 10.1179/135100001101535978 11333117

[B25] Fujishima M. Ibayashi S. Fujii K. Mori S. (1995). Cerebral blood flow and brain function in hypertension. *Hypertens. Res.* 18 111–117. 10.1291/hypres.18.111 7584916

[B26] Fukushima R. Yamazaki E. (2010). Vitamin C requirement in surgical patients. *Curr. Opin. Clin. Nutr. Metab. Care* 13 669–676. 10.1097/MCO.0b013e32833e05bc 20689415

[B27] Garcion E. Wion-Barbot N. Montero-Menei C. N. Berger F. Wion D. (2002). New clues about vitamin D functions in the nervous system. *Trends Endocrinol. Metab.* 13 100–105. 10.1016/S1043-2760(01)00547-1 11893522

[B28] Giavarina D. (2015). Understanding bland altman analysis. *Biochem. Med.* 25 141–151. 10.11613/BM.2015.015 26110027 PMC4470095

[B29] Gómez-Pinilla F. (2008). Brain foods: the effects of nutrients on brain function. *Nat. Rev. Neurosci.* 9 568–578. 10.1038/nrn2421 18568016 PMC2805706

[B30] Grant M. M. Barber V. S. Griffiths H. R. (2005). The presence of ascorbate induces expression of brain derived neurotrophic factor in SH-SY5Y neuroblastoma cells after peroxide insult, which is associated with increased survival. *Proteomics* 5 534–540. 10.1002/pmic.200300924 15627972

[B31] Greger J. Smith S. Snedeker S. (1981). Effect of dietary calcium and phosphorus levels on the utilization of calcium, phosphorus, magnesium, manganese, and selenium by adult males. *Nutr. Res.* 1 315–325. 10.1016/S0271-5317(81)80033-4 7054462

[B32] Grodstein F. Chen J. Willett W. C. (2003). High-dose antioxidant supplements and cognitive function in community-dwelling elderly women. *Am. J. Clin. Nutr.* 77 975–984. 10.1093/ajcn/77.4.975 12663300

[B33] Guo Y.-e Suo N. Cui X. Yuan Q. Xie X. (2018). Vitamin C promotes oligodendrocytes generation and remyelination. *Glia* 66 1302–1316. 10.1002/glia.23306 29423921 PMC6001564

[B34] Gupta P. Tiwari S. Haria J. (2014). Relationship between depression and vitamin C status: a study on rural patients from western uttar pradesh in India. *Int. J. Sci. Study* 1 37–39.

[B35] Hampl J. S. Taylor C. A. Johnston C. S. (2004). Vitamin C deficiency and depletion in the United States: the third national health and nutrition examination survey, 1988 to 1994. *Am. J. Public Health* 94 870–875. 10.2105/AJPH.94.5.870 15117714 PMC1448351

[B36] Hansen S. N. Tveden-Nyborg P. Lykkesfeldt J. (2014). Does vitamin C deficiency affect cognitive development and function? *Nutrients* 6 3818–3846. 10.3390/nu6093818 25244370 PMC4179190

[B37] Harrison F. Allard J. Bixler R. Usoh C. Li L. May J. (2009). Antioxidants and cognitive training interact to affect oxidative stress and memory in APP/PSEN1 mice. *Nutr. Neurosci.* 12 203–218. 10.1179/147683009X423364 19761651 PMC3730134

[B38] Harrison F. E. (2012). A critical review of vitamin C for the prevention of age-related cognitive decline and Alzheimer’s disease. *J. Alzheimers Dis.* 29 711–726. 10.3233/JAD-2012-111853 22366772 PMC3727637

[B39] Harrison F. E. May J. M. (2009). Vitamin C function in the brain: vital role of the ascorbate transporter SVCT2. *Free Radic. Biol. Med.* 46 719–730. 10.1016/j.freeradbiomed.2008.12.018 19162177 PMC2649700

[B40] Healton E. B. Savage D. G. Brust J. C. Garrett T. Lindenbaum J. (1991). Neurologic aspects of cobalamin deficiency. *Medicine* 70 229–245. 10.1097/00005792-199107000-00001 1648656

[B41] Hoffman F. A. (1985). Micronutrient requirements of cancer patients. *Cancer* 55 295–300. 10.1002/1097-0142(19850101)55:1+<295::AID-CNCR2820551315>3.0.CO;2-X 3917362

[B42] Holick M. F. (1996). Vitamin D and bone health. *J. Nutr.* 126(Suppl. 4) 1159S–1164S. 10.1093/jn/126.suppl_4.1159S 8642450

[B43] Hornig D. (1975). Distribution of ascorbic acid, metabolites and analogues in man and animals. *Ann. N. Y. Acad. Sci.* 258 103–118. 10.1111/j.1749-6632.1975.tb29271.x 1106295

[B44] Hsiao P. Y. Mitchell D. Coffman D. Allman R. Locher J. Sawyer P. (2013). Dietary patterns and diet quality among diverse older adults: the university of alabama at birmingham study of aging. *J. Nutr. Health Aging* 17 19–25. 10.1007/s12603-012-0082-4 23299373 PMC3574872

[B45] Institute of Medicine (US) Standing Committee on the Scientific Evaluation of Dietary Reference Intakes and Its Panel on Folate Other B Vitamins and Choline (1998). *Dietary Reference Intakes for Thiamin, Riboflavin, Niacin, Vitamin B6 Folate, Vitamin B12 Pantothenic Acid, Biotin, and Choline.* Washington, DC: National Academies Press. 23193625

[B46] Iqbal K. Khan A. Khattak M. A. K. (2004). Biological significance of ascorbic acid (Vitamin C) in human health–a review. *Pak. J. Nutr.* 3 5–13. 10.3923/pjn.2004.5.13

[B47] Ireland P. Jolley D. Giles G. O’Dea K. Powles J. Rutishauser I. (1994). Development of the Melbourne FFQ: a food frequency questionnaire for use in an Australian prospective study involving an ethnically diverse cohort. *Asia Pac. J. Clin. Nutr.* 3 19–31. 24351203

[B48] IulianoS.OldenA.& WoodsJ. (2013). Meeting the nutritional needs of elderly residents in aged-care: are we doing enough? *J. Nutr. Health Aging* 17 503–508. 10.1007/s12603-013-0042-7 23732545

[B49] Jama J. W. Launer L. J. Witteman J. Den Breeijen J. Breteler M. Grobbee D. (1996). Dietary antioxidants and cognitive function in a population-based sample of older persons the rotterdam study. *Am. J. Epidemiol.* 144 275–280. 10.1093/oxfordjournals.aje.a008922 8686696

[B50] Kallner A. Hartmann D. Hornig D. (1979). Steady-state turnover and body pool of ascorbic acid in man. *Am. J. Clin. Nutr.* 32 530–539. 10.1093/ajcn/32.3.530 420145

[B51] Kang J. H. Cook N. Manson J. Buring J. E. Grodstein F. (2006). A randomized trial of vitamin E supplementation and cognitive function in women. *Arch. Intern. Med.* 166 2462–2468. 10.1001/archinte.166.22.2462 17159011

[B52] Kennedy D. O. (2016). B vitamins and the brain: mechanisms, dose and efficacy—a review. *Nutrients* 8:68. 10.3390/nu8020068 26828517 PMC4772032

[B53] Klinkenberg I. Sambeth A. Blokland A. (2011). Acetylcholine and attention. *Behav. Brain Res.* 221 430–442. 10.1016/j.bbr.2010.11.033 21108972

[B54] Knaack D. Podleski T. (1985). Ascorbic acid mediates acetylcholine receptor increase induced by brain extract on myogenic cells. *Proc. Natl. Acad. Sci. U.S.A.* 82 575–579. 10.1073/pnas.82.2.575 3855568 PMC397083

[B55] Knaack D. Shen I. Salpeter M. M. Podleski T. R. (1986). Selective effects of ascorbic acid on acetylcholine receptor number and distribution. *J. Cell Biol.* 102 795–802. 10.1083/jcb.102.3.795 3949879 PMC2114108

[B56] Kocot J. Luchowska-Kocot D. Kiełczykowska M. Musik I. Kurzepa J. (2017). Does vitamin c influence neurodegenerative diseases and psychiatric disorders? *Nutrients* 9:659. 10.3390/nu9070659 28654017 PMC5537779

[B57] La Rue A. Koehler K. M. Wayne S. J. Chiulli S. J. Haaland K. Y. Garry P. J. (1997). Nutritional status and cognitive functioning in a normally aging sample: a 6-y reassessment. *Am. J. Clin. Nutr.* 65 20–29. 10.1093/ajcn/65.1.20 8988908

[B58] Lecomte E. Herbeth B. Pirollet P. Chancerelle Y. Arnaud J. Musse N. (1994). Effect of alcohol consumption on blood antioxidant nutrients and oxidative stress indicators. *Am. J. Clin. Nutr.* 60 255–261. 10.1093/ajcn/60.2.255 8030604

[B59] Lee D. S. Griffiths B. W. (1985). Human serum vitamin B12 assay methods—a review. *Clin. Biochem.* 18 261–266. 10.1016/S0009-9120(85)80028-X 3902285

[B60] Letenneur L. Proust-Lima C. Le Gouge A. Dartigues J. -F. Barberger-Gateau P. (2007). Flavonoid intake and cognitive decline over a 10-year period. *Am. J. Epidemiol.* 165 1364–1371. 10.1093/aje/kwm036 17369607

[B61] Levine M. Conry-Cantilena C. Wang Y. Welch R. W. Washko P. W. Dhariwal K. R. (1996). Vitamin C pharmacokinetics in healthy volunteers: evidence for a recommended dietary allowance. *Proc. Natl. Acad. Sci. U.S.A.* 93 3704–3709. 10.1073/pnas.93.8.3704 8623000 PMC39676

[B62] Lewin S. (1976). *Vitamin C: Its Molecular Biology and Medical Potential.* London: Academic Press Inc

[B63] Lindeman R. D. Romero L. J. Koehler K. M. Liang H. C. LaRue A. Baumgartner R. N. (2000). Serum vitamin B12, C and folate concentrations in the New Mexico elder health survey: correlations with cognitive and affective functions. *J. Am. Coll. Nutr.* 19 68–76. 10.1080/07315724.2000.10718916 10682878

[B64] Lindenbaum J. Healton E. B. Savage D. G. Brust J. C. Garrett T. J. Podell E. R. (1988). Neuropsychiatric disorders caused by cobalamin deficiency in the absence of anemia or macrocytosis. *New Engl. J. Med.* 318 1720–1728. 10.1056/NEJM198806303182604 3374544

[B65] Madigan S. M. Tracey F. McNulty H. Eaton-Evans J. Coulter J. McCartney H. (1998). Riboflavin and vitamin B-6 intakes and status and biochemical response to riboflavin supplementation in free-living elderly people. *Am. J. Clin. Nutr.* 68 389–395. 10.1093/ajcn/68.2.389 9701198

[B66] Martel J. L. Franklin D. S. (2017). *Vitamin, B1 (Thiamine).* Available at: https://www.ncbi.nlm.nih.gov/books/NBK482360/ (accessed August 12 2018). 29493982

[B67] Massee L. A. Ried K. Pase M. Travica N. Yoganathan J. Scholey A. (2015). The acute and sub-chronic effects of cocoa flavanols on mood, cognitive and cardiovascular health in young healthy adults: a randomized, controlled trial. *Front. Pharmacol.* 6:93. 10.3389/fphar.2015.00093 26042037 PMC4438591

[B68] May J. M. Li L. Hayslett K. Qu Z. -C. (2006). Ascorbate transport and recycling by SH-SY5Y neuroblastoma cells: response to glutamate toxicity. *Neurochem. Res.* 31 785–794. 10.1007/s11064-006-9077-z 16791474

[B69] McAfoose J. Baune B. (2009). Evidence for a cytokine model of cognitive function. *Neurosci. Biobehav. Rev.* 33 355–366. 10.1016/j.neubiorev.2008.10.005 18996146

[B70] Mendelson S. D. (2008). *10-Nutritional Supplements and Metabolic Syndrome. Metabolic Syndrome and Psychiatric Illness.* San Diego, CA: Academic Press, 141–186.

[B71] Mollin D. Ross G. (1952). The vitamin B12 concentrations of serum and urine of normals and of patients with megaloblastic anaemias and other diseases. *J. Clin. Pathol.* 5 129–139. 10.1136/jcp.5.2.129 14938452 PMC1023543

[B72] National Health and Medical Research Council (2009). *Australian Guidelines to Reduce Health Risks From Drinking Alcohol.* Canberra: NHMRC.

[B73] Newton H. M. Schorah C. Habibzadeh N. Morgan D. Hullin R. (1985). The cause and correction of low blood vitamin C concentrations in the elderly. *Am. J. Clin. Nutr.* 42 656–659. 10.1093/ajcn/42.4.656 4050725

[B74] Noble M. Healey C. S. McDougal-Chukwumah L. D. Brown T. M. (2013). Old disease, new look? A first report of parkinsonism due to scurvy, and of refeeding-induced worsening of scurvy. *Psychosomatics* 54 277–283. 10.1016/j.psym.2013.02.001 23473448

[B75] Nooyens A. C. Milder I. E. Van Gelder B. M. Bueno-de-Mesquita H. B. Van Boxtel M. P. Verschuren W. M. (2015). Diet and cognitive decline at middle age: the role of antioxidants. *Br. J. Nutr.* 113 1410–1417. 10.1017/S0007114515000720 25851267

[B76] O’Keefe C. A. Bailey L. B. Thomas E. A. Hofler S. A. Davis B. A. Cerda J. J. (1995). Controlled dietary folate affects folate status in nonpregnant women. *J. Nutr.* 125 2717–2725. 7562109 10.1093/jn/125.10.2717

[B77] Ortega R. M. Requejo A. M. Andrés P. López-Sobaler A. M. Quintas M. E. Redondo M. R. (1997). Dietary intake and cognitive function in a group of elderly people. *Am. J. Clin. Nutr.* 66 803–809. 10.1093/ajcn/66.4.803 9322553

[B78] Padayatty S. J. Doppman J. L. Chang R. Wang Y. Gill J. Papanicolaou D. A. (2007). Human adrenal glands secrete vitamin C in response to adrenocorticotrophic hormone. *Am. J. Clin. Nutr.* 86 145–149. 10.1093/ajcn/86.1.145 17616774

[B79] Padayatty S. J. Sun H. Wang Y. Riordan H. D. Hewitt S. M. Katz A. (2004). Vitamin C pharmacokinetics: implications for oral and intravenous use. *Ann. Intern. Med.* 140 533–537. 10.7326/0003-4819-140-7-200404060-00010 15068981

[B80] Peacock J. M. Folsom A. R. Knopman D. S. Mosley T. H. Goff D. C. Szklo M. (2000). Dietary antioxidant intake and cognitive performance in middle-aged adults. *Public Health Nutr.* 3 337–343. 10.1017/S1368980000000380 10980106

[B81] Pearson J. F. Pullar J. M. Wilson R. Spittlehouse J. K. Vissers M. Skidmore P. M. (2017). Vitamin C status correlates with markers of metabolic and cognitive health in 50-year-olds: findings of the CHALICE cohort study. *Nutrients* 9:831. 10.3390/nu9080831 28771190 PMC5579624

[B82] Péneau S. Galan P. Jeandel C. Ferry M. Andreeva V. Hercberg S. (2011). Fruit and vegetable intake and cognitive function in the SU. VI. MAX 2 prospective study. *Am. J. Clin. Nutr.* 94 1295–1303. 10.3945/ajcn.111.014712 21955649

[B83] Perrig W. J. Perrig P. Stähelin H. (1997). The relation between antioxidants and memory performance in the old and very old. *J. Am. Geriatr. Soc.* 45 718–724. 10.1111/j.1532-5415.1997.tb01476.x 9180666

[B84] Pipingas A. Harris E. Tournier E. King R. Kras M. Stough C. K. (2010). Assessing the efficacy of nutraceutical interventions on cognitive functioning in the elderly. *Curr. Top. Nutraceutical Res.* 8 79–88.

[B85] Poly C. Massaro J. M. Seshadri S. Wolf P. A. Cho E. Krall E. (2011). The relation of dietary choline to cognitive performance and white-matter hyperintensity in the Framingham Offspring Cohort. *Am. J. Clin. Nutr.* 94 1584–1591. 10.3945/ajcn.110.008938 22071706 PMC3252552

[B86] Quinn J. Suh J. Moore M. M. Kaye J. Frei B. (2003). Antioxidants in Alzheimer’s disease-vitamin C delivery to a demanding brain. *J. Alzheimers Dis.* 5 309–313. 10.3233/JAD-2003-5406 14624026

[B87] Reay J. Smith M. Riby L. (2013). B vitamins and cognitive performance in older adults: review. *ISRN Nutr.* 2013:650983. 10.5402/2013/650983 24959550 PMC4045270

[B88] Rebec G. V. Pierce R. C. (1994). A vitamin as neuromodulator: ascorbate release into the extracellular fluid of the brain regulates dopaminergic and glutamatergic transmission. *Prog. Neurobiol.* 43 537–565. 10.1016/0301-0082(94)90052-3 7816935

[B89] Rebouche C. J. (1991). Ascorbic acid and carnitine biosynthesis. *Am. J. Clin. Nutr.* 54 1147S–1152S. 10.1093/ajcn/54.6.1147s 1962562

[B90] Revel F. Gilbert T. Roche S. Drai J. Blond E. Ecochard R. (2015). Influence of oxidative stress biomarkers on cognitive decline. *J. Alzheimers Dis.* 45 553–560. 10.3233/JAD-141797 25589716

[B91] Riggs K. M. Spiro A. Tucker K. Rush D. (1996). Relations of vitamin B-12, vitamin B-6, folate, and homocysteine to cognitive performance in the Normative Aging Study. *Am. J. Clin. Nutr.* 63 306–314. 10.1093/ajcn/63.3.306 8602585

[B92] Rizvi S. I. Pandey K. B. Jha R. Maurya P. K. (2009). Ascorbate recycling by erythrocytes during aging in humans. *Rejuvenation Res.* 12 3–6. 10.1089/rej.2008.0787 19072252

[B93] Sato R. Helzlsouer K. Comstock G. Hoffman S. (2006). A cross-sectional study of vitamin C and cognitive function in older adults: the differential effects of gender. *J. Nutr. Health Aging* 10 37–44. 16453056

[B94] Savage D. G. Lindenbaum J. (1995). 11 Neurological complications of acquired cobalamin deficiency: clinical aspects. *Baillières Clin. Haematol.* 8 657–678. 10.1016/S0950-3536(05)80225-2 8534966

[B95] Schleicher R. L. Carroll M. D. Ford E. S. Lacher D. A. (2009). Serum vitamin C and the prevalence of vitamin C deficiency in the united states: 2003–2004 national health and nutrition examination survey (NHANES). *Am. J. Clin. Nutr.* 90 1252–1263. 10.3945/ajcn.2008.27016 19675106

[B96] Sheridan L. K. Fitzgerald H. E. Adams K. M. Nigg J. T. Martel M. M. Puttler L. I. (2006). Normative symbol digit modalities test performance in a community-based sample. *Arch. Clin. Neuropsychol.* 21 23–28. 10.1016/j.acn.2005.07.003 16139470

[B97] Sindi S. Juster R. -P. Wan N. Nair N. Ying Kin N. Lupien S. (2012). Depressive symptoms, cortisol, and cognition during human aging: the role of negative aging perceptions. *Stress* 15 130–137. 10.3109/10253890.2011.599047 21801079

[B98] Singer J. Trollor J. N. Baune B. T. Sachdev P. S. Smith E. (2014). Arterial stiffness, the brain and cognition: a systematic review. *Ageing Res. Rev.* 15 16–27. 10.1016/j.arr.2014.02.002 24548924

[B99] Slutsky I. Abumaria N. Wu L. -J. Huang C. Zhang L. Li B. (2010). Enhancement of learning and memory by elevating brain magnesium. *Neuron* 65 165–177. 10.1016/j.neuron.2009.12.026 20152124

[B100] Smith P. F. Smith A. Miners J. McNeil J. Proudfoot A. (2000). *The safety aspects of dietary caffeine. Australia: Report from the Expert Working group, 202–223.* Available at: https://www.foodstandards.gov.au/publications/Documents/safety%20aspects%20of%20dietary%20caffeine.pdf (accessed August 06 2018).

[B101] Sotiriou S. Gispert S. Cheng J. Wang Y. Chen A. Hoogstraten-Miller S. (2002). Ascorbic-acid transporter Slc23a1 is essential for vitamin C transport into the brain and for perinatal survival. *Nat. Med.* 8 514–517. 10.1038/0502-514 11984597

[B102] Spector R. (1977). Vitamin homeostasis in the central nervous system. *New Engl. J. Med.* 296 1393–1398. 10.1056/NEJM197706162962409 323714

[B103] Spector R. Lorenzo A. (1973). Ascorbic acid homeostasis in the central nervous system. *Am. J. Physiol. Leg. Content* 225 757–763. 10.1152/ajplegacy.1973.225.4.757 4743366

[B104] Starns J. J. Ratcliff R. (2010). The effects of aging on the speed–accuracy compromise: boundary optimality in the diffusion model. *Psychol. Aging* 25 377–390. 10.1037/a0018022 20545422 PMC2896207

[B105] Swain D. P. Franklin B. A. (2006). Comparison of cardioprotective benefits of vigorous versus moderate intensity aerobic exercise. *Am. J. Cardiol.* 97 141–147. 10.1016/j.amjcard.2005.07.130 16377300

[B106] Tombaugh T. N. McIntyre N. J. (1992). The mini-mental state examination: a comprehensive review. *J. Am. Geriatr. Soc.* 40 922–935. 10.1111/j.1532-5415.1992.tb01992.x 1512391

[B107] Torda C. Wolff H. G. (1945). Effect of vitamins on acetylcholine synthesis. The apparently specific action of Vitamin E. *Proc. Soc. Exp. Biol. Med.* 58 163–165. 10.3181/00379727-58-14882

[B108] Travica N. Ried K. Sali A. Scholey A. Hudson I. Pipingas A. (2017). Vitamin C status and cognitive function: a systematic review. *Nutrients* 9:960. 10.3390/nu9090960 28867798 PMC5622720

[B109] Trumbo P. Schlicker S. Yates A. A. Poos M. (2002). Dietary reference intakes for energy, carbohydrate, fiber, fat, fatty acids, cholesterol, protein and amino acids. *J. Am. Dietetic Assoc.* 102 1621–1630. 10.1016/S0002-8223(02)90346-9 12449285

[B110] Trumbo P. Yates A. A. Schlicker S. Poos M. (2001). Dietary reference intakes: vitamin A, vitamin K, arsenic, boron, chromium, copper, iodine, iron, manganese, molybdenum, nickel, silicon, vanadium, and zinc. *J. Am. Dietetic Assoc.* 101 294–301. 10.1016/S0002-8223(01)00078-5 11269606

[B111] Tsao C. W. Seshadri S. Beiser A. S. Westwood A. J. DeCarli C. Au R. (2013). Relations of arterial stiffness and endothelial function to brain aging in the community. *Neurology* 81 984–991. 10.1212/WNL.0b013e3182a43e1c 23935179 PMC3888200

[B112] Uttara B. Singh A. V. Zamboni P. Mahajan R. (2009). Oxidative stress and neurodegenerative diseases: a review of upstream and downstream antioxidant therapeutic options. *Curr. Neuropharmacol.* 7 65–74. 10.2174/157015909787602823 19721819 PMC2724665

[B113] Vauzour D. (2012). Dietary polyphenols as modulators of brain functions: biological actions and molecular mechanisms underpinning their beneficial effects. *Oxid. Med. Cell. Longev.* 2012:914273 10.1155/2012/914273 22701758 PMC3372091

[B114] Ward M. S. Lamb J. May J. M. Harrison F. E. (2013). Behavioral and monoamine changes following severe vitamin C deficiency. *J. Neurochem.* 124 363–375. 10.1111/jnc.12069 23106783 PMC3540126

[B115] Weinstein M. Babyn P. Zlotkin S. (2001). An orange a day keeps the doctor away: scurvy in the year 2000. *Pediatrics* 108:e55. 10.1542/peds.108.3.e55 11533373

[B116] White H. L. Scates P. W. (1990). Acetyl-L-carnitine as a precursor of acetylcholine. *Neurochem. Res.* 15 597–601. 10.1007/BF00973749 2215852

[B117] Zhang S. Hunter D. J. Forman M. R. Rosner B. A. Speizer F. E. Colditz G. A. (1999). Dietary carotenoids and vitamins A, C, and E and risk of breast cancer. *J. Nat. Cancer Inst.* 91 547–556. 10.1093/jnci/91.6.547 10088626

