# Supplementary material for: Plasma Vitamin C Concentrations and Cognitive Function: A Cross-Sectional Study
Source: Front Aging Neurosci. 2019 Apr 2;11:72. doi: 10.3389/fnagi.2019.00072 (PMC6454201; doi:10.3389/fnagi.2019.00072)
Supplement: Supplementary file 1 [file Table_1.DOCX]

Supplementary Table 1. Comparison between self-reported vitamin C supplementers and non-vitamin C supplementers in the adequate plasma vitamin C group on the HVLT-R cognitive measures and SDMT score.

| **Paper and pen cognitive assessment** | **Mean score ± SE (n = 80)** | | **Covariates** | **Parameter Estimates** | | | **Differences between Adequate vs deficient vitamin C level groups** | | |
| --- | --- | --- | --- | --- | --- | --- | --- | --- | --- |
| Adequate vitamin C plasma groups | Adequate  (n = 47) | Adequate supplementers  (n = 20) |  | B | SE | p-value | M | SE | p-value |
| *HVLT-R* |  | | | | | | | | |
| Trial 1 | 7.60 ± 1.78 | 7.35 ± 1.66 | None |  |  |  | 0.25 | 0.47 | 0.60 |
| Trial 2 | 9.72 ± 1.68 | 9.95 ± 2.11 | None |  |  |  | 0.23 | 0.49 | 0.64 |
| Trial 3 | 10.47 ± 1.46 | 11.15 ± 1.09 | None |  |  |  | 0.68 | 0.36 | 0.007 |
| Delayed recall | 9.68 ± 0.27 | 10.00 ± 0.43 | Age | -0.036 | 0.016 | 0.024 | 0.33 | 0.52 | 0.54 |
| Total recall | 27.85 ± 0.59 | 28.40 ± 0.91 | None |  |  |  | 0.55 | 1.01 | 0.62 |
| Recognition index | 11.42 ± 0.17 | 11.32 ± 0.27 | Age | -0.024 | 0.010 | 0.013 | 0.12 | 0.32 | 0.72 |
| SDMT | 50.65 ± 1.23 | 47.58 ± 1.95 | Age | -0.37 | 0.07 | < 0.001 | 3.07 | 2.37 | 0.20 |

SE = Standard Error, B = Beta value, HVLT-R = Hopkins Verbal Learning Test- Revised, SDMT = Symbol Digits Modalities Test
