# Supplementary material for: Plasma Vitamin C Concentrations and Cognitive Function: A Cross-Sectional Study
Source: Front Aging Neurosci. 2019 Apr 2;11:72. doi: 10.3389/fnagi.2019.00072 (PMC6454201; doi:10.3389/fnagi.2019.00072)
Supplement: Supplementary file 2 [file Table_2.docx]

| **SUCCAB Task**  **(Reaction time)** | **Mean score ± SE (n = 80)** | | **Covariates** | **Parameter Estimates** | | | **Differences between Adequate vs deficient vitamin C level groups** | | |
| --- | --- | --- | --- | --- | --- | --- | --- | --- | --- |
| Adequate vitamin C plasma groups | Adequate  (n = 47) | Adequate supplementers  (n = 20) |  | B | SE | p- value | Mean | SE | p-value |
| Simple reaction time | 325.72 ± 11.97 | 326.27 ± 18.35 | None |  |  |  | 0.55 | 21.90 | 0.98 |
| Choice reaction time | 540.05 ± 16.98 | 543.72 ± 26.04 | None |  |  |  | 3.68 | 31.09 | 0.91 |
| Immediate recognition memory | 1009.50 ± 42.25 | 1121.38 ± 64.77 | None |  |  |  | 111.88 | 77.33 | 0.15 |
| Congruent Stroop | 749.89 ± 160.73 | 786.90 ± 125.63 | None |  |  |  | 37.01 | 40.40 | 0.36 |
| Incongruent Stroop | 901.58 ± 32.03 | 959.69 ± 50.65 | None |  |  |  | 58.11 | 59.93 | 0.34 |
| Spatial working memory | 1070.16 ± 46.10 | 1048.03 ± 70.66 | None |  |  |  | 22.13 | 84.37 | 0.79 |
| Contextual memory | 1046.84 ± 32.36 | 1143.21 ± 50.79 | Number of meds | 33.55 | 16.07 | 0.04 | 96.38 | 60.73 | 0.12 |
|  |  |  | Years of education | 44.52 | 17.73 | 0.015 |  |  |  |
| Delayed recognition memory | 1030.65 ± 36.36 | 1080.44 ± 55.15 | None |  |  |  | 59.79 | 66.06 | 0.37 |

Supplementary Table 2. Comparison between self-reported vitamin C supplementers and non-vitamin C supplementers in the adequate plasma vitamin C group on the SUCCAB task reaction time.

Meds = Medications, SE = Standard Error, B = Beta value, SUCCAB = Swinburne University Computerized Cognitive Battery
