# Supplementary material for: Plasma Vitamin C Concentrations and Cognitive Function: A Cross-Sectional Study
Source: Front Aging Neurosci. 2019 Apr 2;11:72. doi: 10.3389/fnagi.2019.00072 (PMC6454201; doi:10.3389/fnagi.2019.00072)
Supplement: Supplementary file 3 [file Table_3.DOCX]

| **SUCCAB Task**  **(Ratio)** | **Mean score ± SE (n = 80)** | | **Covariates** | **Parameter Estimates** | | | **Differences between Adequate vs deficient vitamin C level groups** | | |
| --- | --- | --- | --- | --- | --- | --- | --- | --- | --- |
| Adequate vitamin C plasma groups | Adequate  (n = 47) | Adequate supplementers  (n = 20) |  | B | SE | p- value | Mean | SE | p-value |
| Simple reaction time | 322.93 ± 8.66 | 315.08 ± 13.27 | None |  |  |  | 7.85 | 15.84 | 0.62 |
| Choice reaction time | 189.28 ± 4.81 | 183.41 ± 7.38 | None |  |  |  | 5.87 | 8.81 | 0.51 |
| Immediate recognition memory | 83.96 ± 3.09 | 75.61 ± 4.73 | None |  |  |  | 8.35 | 5.65 | 0.14 |
| Congruent stroop | 135.50 ± 3.79 | 132.00 ± 5.93 | Age | -0.456 | 0.217 | 0.04 | 3.50 | 7.26 | 0.63 |
| Incongruent stroop | 109.79 ± 3.52 | 110.55 ± 5.70 | Age | -0.605 | 0.201 | 0.004 | 0.76 | 6.85 | 0.91 |
| Spatial working memory | 80.13 ± 3.82 | 83.14 ± 6.01 | Age | -0.614 | 0.218 | 0.006 | 3.008 | 7.338 | 0.68 |
| Contextual memory | 80.45 ± 3.71 | 64.43 ± 5.77 | None |  |  |  | 16.02 | 6.86 | 0.023* |
| Delayed recognition memory | 73.01 ± 2.34 | 69.39 ± 3.54 | None |  |  |  | 3.62 | 4.24 | 0.40 |

Supplementary Table 3. Comparison between self-reported vitamin C supplementers and non-vitamin C supplementers in the adequate plasma vitamin C group on the SUCCAB ratio performance.

* p < 0.05, SE = Standard Error, B = Beta value, SUCCAB = Swinburne University Computerized Cognitive Battery
