# Supplementary material for: Plasma Vitamin C Concentrations and Cognitive Function: A Cross-Sectional Study
Source: Front Aging Neurosci. 2019 Apr 2;11:72. doi: 10.3389/fnagi.2019.00072 (PMC6454201; doi:10.3389/fnagi.2019.00072)
Supplement: Supplementary file 4 [file Data_Sheet_1.pdf]

Supplementary Figure 1
Plasma Vitamin C levels HPLC vs Colorimetric

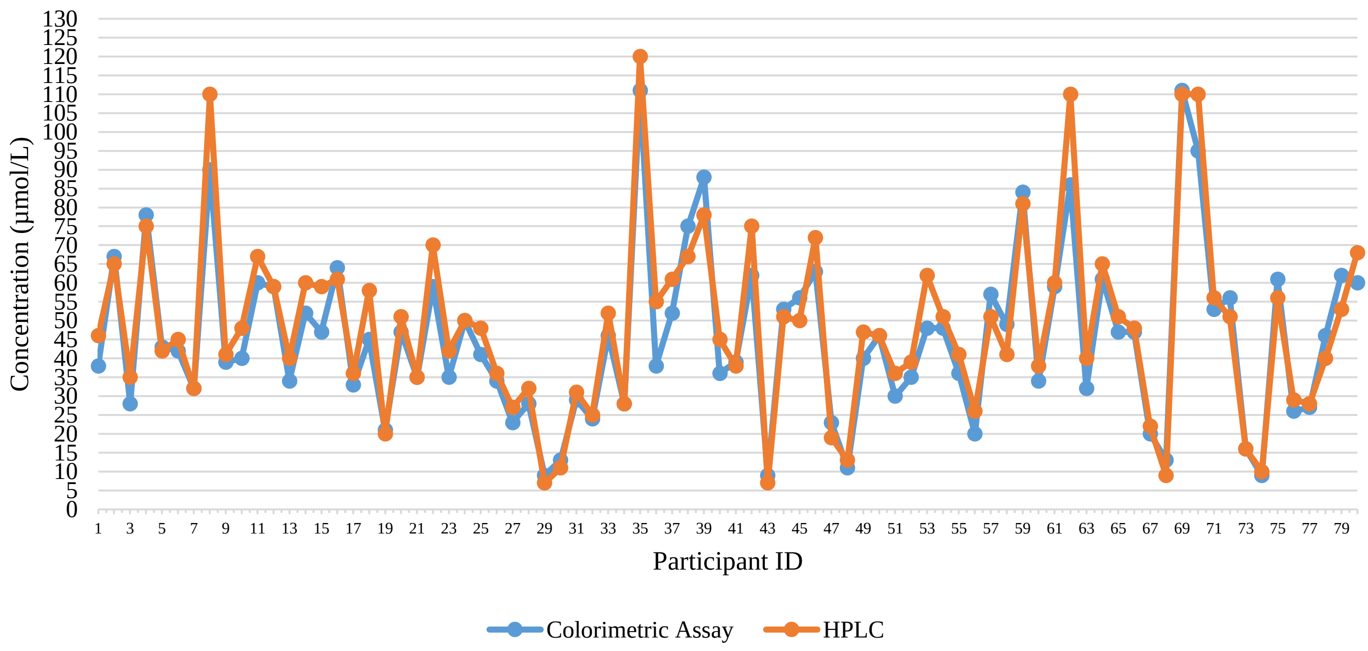

Legend: Mean plasma vitamin C concentrations compared between HPLC (M ± SD = 45.69 ± 22.14 μmol/L) and colorimetric (M ± SD = 48.53 ± 23.90 μmol/L) biochemical analyses, HPLC = High Performance Liquid Chromatography, M = Mean, SD = Standard Deviation.
