# Supplementary material for: Plasma Vitamin C Concentrations and Cognitive Function: A Cross-Sectional Study
Source: Front Aging Neurosci. 2019 Apr 2;11:72. doi: 10.3389/fnagi.2019.00072 (PMC6454201; doi:10.3389/fnagi.2019.00072)
Supplement: Supplementary file 5 [file Data_Sheet_2.PDF]

Supplementary Figure 2. Bland-Altman Plot comparing Colorimetric and HPLC plasma vitamin C

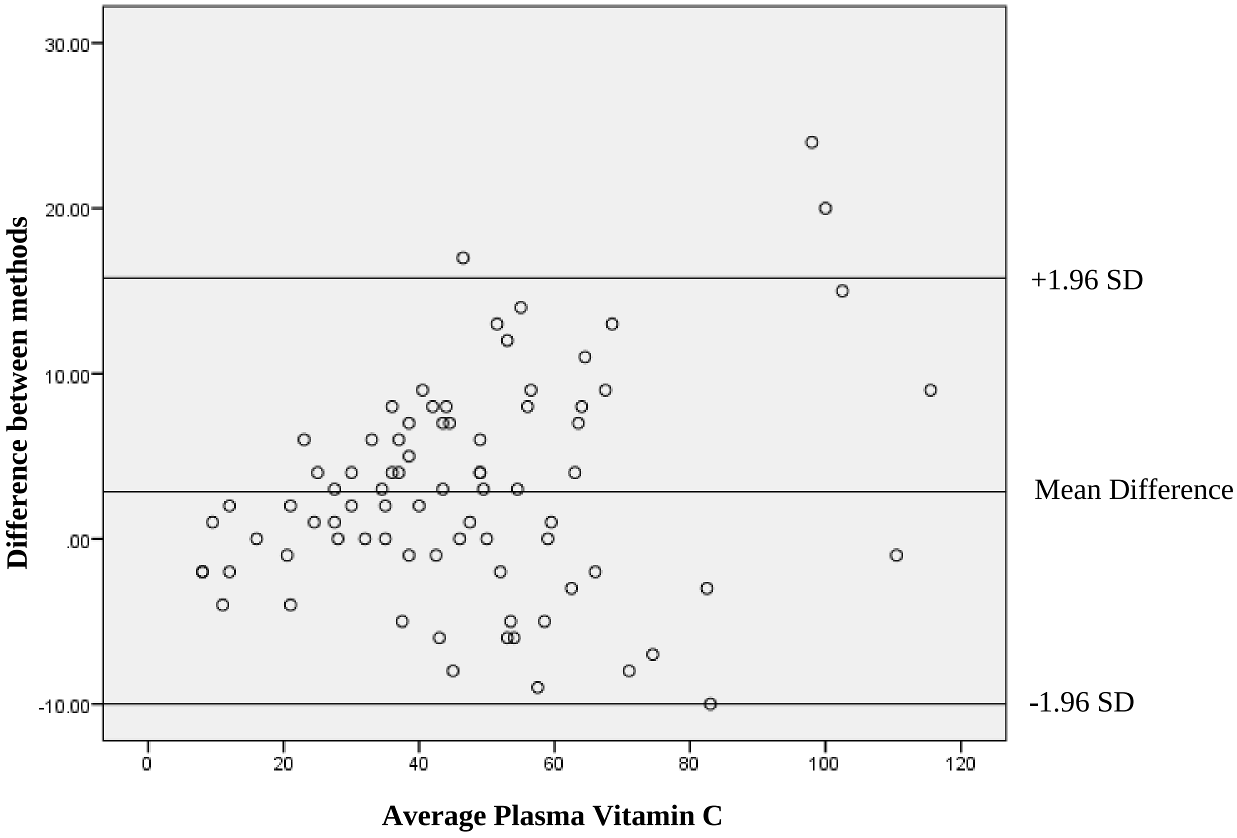

Legend: Plot demonstrates that comparability between HPLC and colorimetric assays was achieved given 95% of the data points were within ± 2 standard deviations (upper and lower lines) of the mean (middle line) difference, SD = Standard Deviation
