# Supplementary material for: Plasma Vitamin C Concentrations and Cognitive Function: A Cross-Sectional Study
Source: Front Aging Neurosci. 2019 Apr 2;11:72. doi: 10.3389/fnagi.2019.00072 (PMC6454201; doi:10.3389/fnagi.2019.00072)
Supplement: Supplementary file 6 [file Data_Sheet_3.PDF]

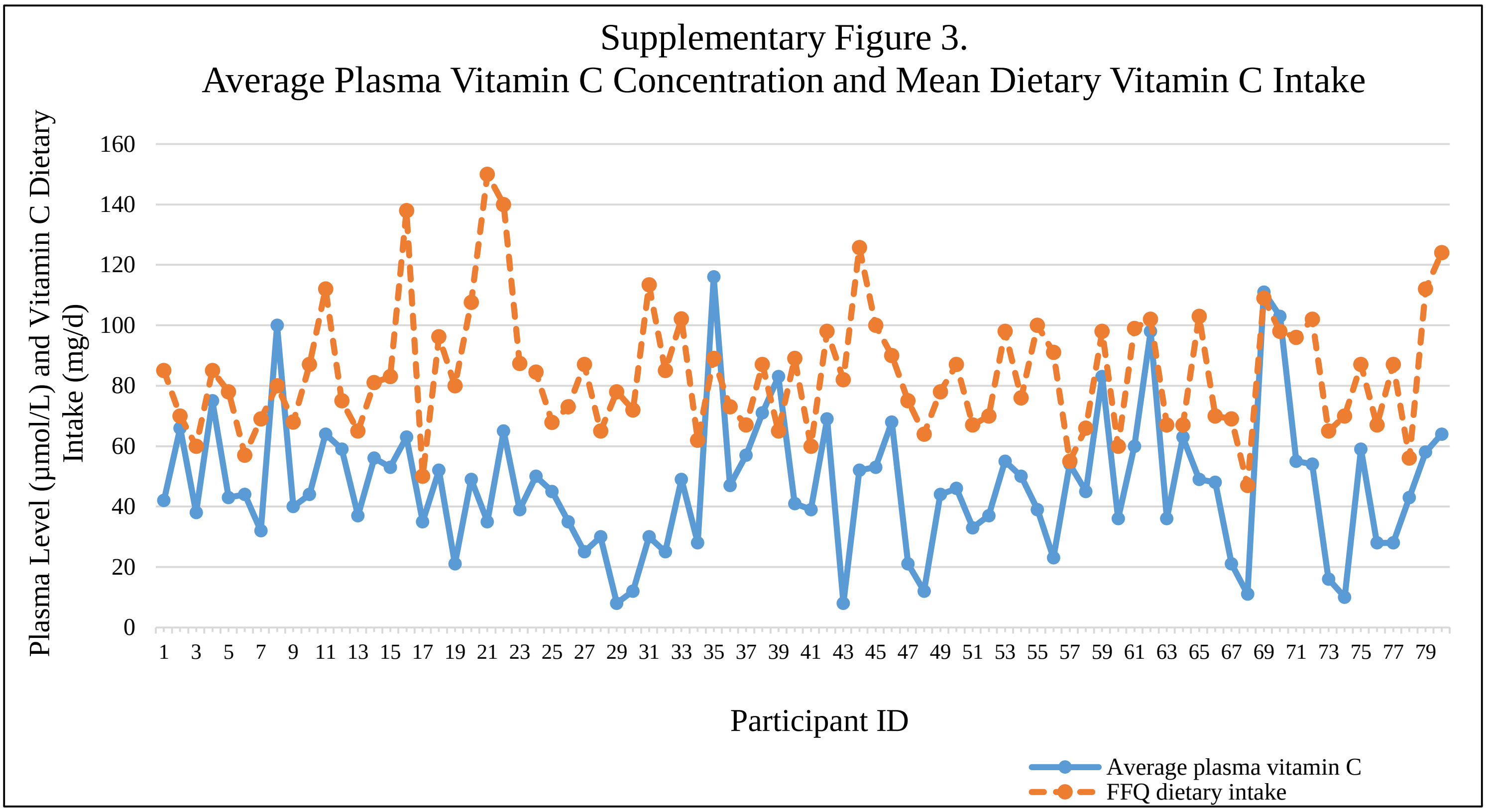

Legend: Plasma vitamin C concentrations compared with mean daily dietary vitamin C intake, Spearman correlation: $r_s\,(80) = 0.438$, $p < 0.001$, µmol/L = micromole per litre, mg/d = milligram per day.
